# Predicting evolutionary targets and parameters of gene deletion from expression data

**DOI:** 10.1093/bioadv/vbae002

**Published:** 2024-01-17

**Authors:** Andre Luiz Campelo dos Santos, Michael DeGiorgio, Raquel Assis

**Affiliations:** Department of Electrical Engineering and Computer Science, Florida Atlantic University, Boca Raton, FL 33431, United States; Department of Electrical Engineering and Computer Science, Florida Atlantic University, Boca Raton, FL 33431, United States; Department of Electrical Engineering and Computer Science, Florida Atlantic University, Boca Raton, FL 33431, United States; Institute for Human Health and Disease Intervention, Florida Atlantic University, Boca Raton, FL 33431, United States

## Abstract

**Motivation:**

Gene deletion is traditionally thought of as a nonadaptive process that removes functional redundancy from genomes, such that it generally receives less attention than duplication in evolutionary turnover studies. Yet, mounting evidence suggests that deletion may promote adaptation via the “less-is-more” evolutionary hypothesis, as it often targets genes harboring unique sequences, expression profiles, and molecular functions. Hence, predicting the relative prevalence of redundant and unique functions among genes targeted by deletion, as well as the parameters underlying their evolution, can shed light on the role of gene deletion in adaptation.

**Results:**

Here, we present CLOUDe, a suite of machine learning methods for predicting evolutionary targets of gene deletion events from expression data. Specifically, CLOUDe models expression evolution as an Ornstein–Uhlenbeck process, and uses multi-layer neural network, extreme gradient boosting, random forest, and support vector machine architectures to predict whether deleted genes are “redundant” or “unique”, as well as several parameters underlying their evolution. We show that CLOUDe boasts high power and accuracy in differentiating between classes, and high accuracy and precision in estimating evolutionary parameters, with optimal performance achieved by its neural network architecture. Application of CLOUDe to empirical data from *Drosophila* suggests that deletion primarily targets genes with unique functions, with further analysis showing these functions to be enriched for protein deubiquitination. Thus, CLOUDe represents a key advance in learning about the role of gene deletion in functional evolution and adaptation.

**Availability and implementation:**

CLOUDe is freely available on GitHub (https://github.com/anddssan/CLOUDe).

## 1 Introduction

Gene deletion is a mutational process that primarily affects members of multi-copy gene families (Albalat and Cañestro 2016). Thus, gene duplication and deletion are naturally intertwined, together contributing to evolutionary turnover that drives divergence and speciation (Zhang 2003, Albalat and Cañestro 2016). In particular, gene duplication produces two copies of an ancestral gene, both of which may be evolutionarily retained through mechanisms that either result in their functional redundancy (conservation; Ohno 1970, Zhang 2003) or uniqueness (neofunctionalization, subfunctionalization, or both; Ohno 1970, Force *et al.* 1999, Stoltzfus 1999, Zhang 2003, He and Zhang 2005, Rastogi and Liberles 2005). Traditionally, deletion is thought of as a nonadaptive process that rids genomes of functional redundancy, such that it generally receives less attention than duplication in evolutionary turnover studies (Albalat and Cañestro 2016, Assis 2019). Yet, mounting evidence suggests that deletion may promote adaptation via the “less-is-more” evolutionary hypothesis (Olson 1999), as it often targets genes harboring unique sequences, expression profiles, and molecular functions (Hottes *et al.* 2013, Kvitek and Sherlock 2013, Albalat and Cañestro 2016, Assis 2019). Hence, determining the relative prevalence of redundant and unique functions among deleted genes, as well as the parameters underlying their evolution, can shed light on the role of deletion in adaptation.

Though several studies have investigated the adaptive significance of deleted genes (Hottes *et al.* 2013, Kvitek and Sherlock 2013, Albalat and Cañestro 2016, Assis 2019), there are currently no methods for predicting their functional redundancy or underlying evolutionary parameters. However, DeGiorgio and Assis (2021) recently developed an analogous method for gene duplication, CLOUD (CLassification using Ornstein–Uhlenbeck of Duplications), which uses expression data from two species to predict the evolutionary mechanisms and parameters involved in the retention of duplicate genes. Specifically, CLOUD first models expression evolution after gene duplication along a phylogeny relating the two species as an Ornstein–Uhlenbeck (OU) process (DeGiorgio and Assis 2021), an extension of the Brownian motion random walk that is constrained by a constant pull toward an optimum (Martins 1994). Hence, random drift is represented by Brownian motion, natural selection by pull, and fittest phenotype by the optimum (Hansen 1997, Butler and King 2004). Then, CLOUD uses a multi-layer neural network architecture to predict evolutionary retention mechanisms and parameters of duplicate genes (DeGiorgio and Assis 2021). Recently, a similar machine learning framework, PiXi (PredIcting eXpression dIvergence), was designed for predicting expression divergence and expression optima of single-copy genes in two species (Piya *et al.* 2023). PiXi also models expression evolution as an OU process and uses a multi-layer neural network, as well as two additional machine learning architectures—random forest and support vector machine—for making predictions (Piya *et al.* 2023). Encouragingly, both CLOUD (DeGiorgio and Assis 2021) and PiXi (Piya *et al.* 2023) demonstrate high predictive power and accuracy and also globally outperform alternative distance-based methods (Perry and Assis 2016, Piya *et al.* 2023), highlighting the utility of leveraging machine learning for these types of evolutionary questions.

With this in mind, we present CLassification using Ornstein–Uhlenbeck of Deletions (CLOUDe). Similar to CLOUD (DeGiorgio and Assis 2021) and PiXi (Piya *et al.* 2023), CLOUDe models expression evolution after gene deletion along a phylogeny relating two sister species as an OU process. As with PiXi (Piya *et al.* 2023), the output of these models is fed to predictors composing multi-layer neural network, random forest, and support vector machine architectures—in addition to a newly implemented extreme gradient boosting architecture—which classify deleted genes as either “redundant” or “unique” and estimate several parameters driving their evolution. We show through an array of simulations that CLOUDe achieves high power and accuracy in differentiating between “redundant” and “unique” classes, as well as high accuracy and precision in estimating evolutionary parameters. Further, in contrast to the evolutionary scenarios examined by CLOUD (DeGiorgio and Assis 2021) and PiXi (Piya *et al.* 2023), here we apply maximum likelihood models based on OU evolution and demonstrate the superior predictive ability of CLOUDe. Application of the CLOUDe neural network predictor to empirical data from *Drosophila* (Assis 2019) indicates that most deleted genes possess unique gene expression profiles, and that evolution after deletion is driven by a combination of neutral and selective forces, together supporting the hypothesis that gene deletion can often be adaptive. CLOUDe has been implemented as an open-source R package and is available with instructions and an example dataset at https://github.com/anddssan/CLOUDe. Its input data can include gene expression measured for a single or multiple conditions of varying types, such as tissues or developmental stages, making it applicable to a wide range of unicellular and multicellular organisms.

## 2 Methods

### 2.1 Development of the CLOUDe predictors

**Figure 1.**
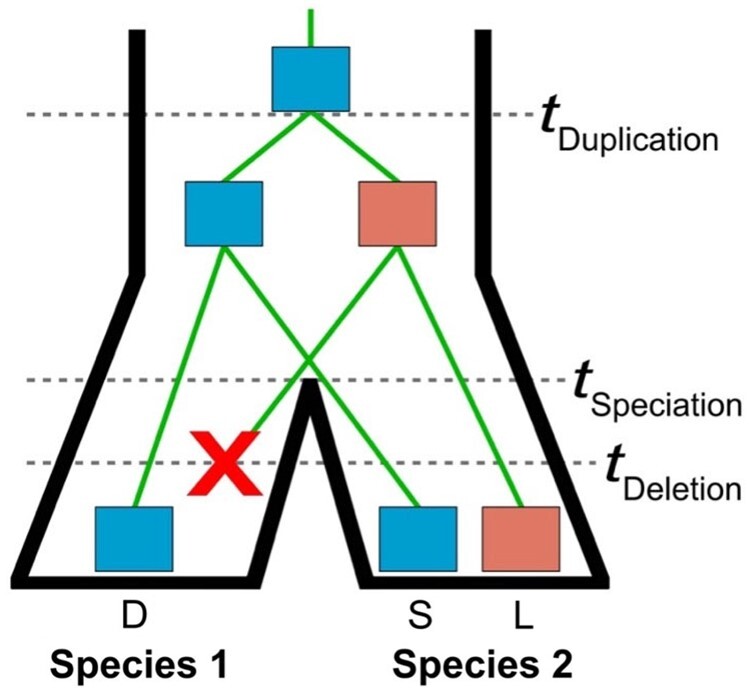
Schematic of the deletion scenario considered in this study. Depicted is the relationship between two species (black outer phylogeny) and their genes (green inner phylogeny). At time tDuplication, a gene (blue) underwent a duplication event, resulting in a pair of duplicate genes in the ancestral lineage. At time tSpeciation, a speciation event led to the emergence of the Species 1 and Species 2 lineages. At time tDeletion, the pair of duplicate genes underwent a deletion event, resulting in the loss of one gene copy in the lineage of Species 1 (red cross). Here, the single-copy gene in Species 1 is denoted as D, the ortholog of this gene in Species 2 as S, and the gene present in Species 2 that was deleted in Species 1 as L. Note that either duplicate gene copy can be deleted, and both possibilities are considered in this study.

Here we consider the scenario in which duplication created two gene copies in the common ancestor of a pair of related species, Species 1 and Species 2, and subsequent deletion resulted in the loss of one of these gene copies in the lineage of Species 1 (Fig. 1). Thus, Species 1 represents the derived state and carries one gene copy, whereas Species 2 represents the ancestral state and harbors both gene copies. We designate the gene in Species 1 as “derived” (D), the ortholog of this gene in Species 2 as “survived” (S), and the gene present in Species 2 that was deleted in Species 1 as “lost” (L). Additionally, let $\theta_{1}$ denote the expression optimum for D and S genes. Similarly, let $\theta_{2}$ denote the expression optimum for the L gene, for the duplicate genes immediately after duplication in the ancestor, and for the single-copy gene prior to duplication in the ancestor. We thus assume that at least one of the duplicate gene copies retained the expression optimum from the ancestral copy, as this assumption is supported by empirical findings in several diverse taxa (Assis and Bachtrog 2013, Assis and Bachtrog 2015, Chau and Goodisman 2017, Jiang and Assis 2019). We then adapt the OU framework to model expression evolution along a phylogeny relating the D, S, and L genes, with random changes occurring through phenotypic drift with strength $\sigma^{2}$, and changes toward expression optima $\theta_{1}$ and $\theta_{2}$ through selection with strength α. For each deletion, we seek to predict whether the ancestral duplicate gene targets were functionally “redundant” ($\theta_{1}=\theta_{2}$) or “unique” ($\theta_{1}\neq \theta_{2}$), as well as $\theta_{1}$, $\theta_{2}$, and the relative strength of drift to selection $\log_{10}\left(\sigma^{2}/(2\alpha )\right)$, i.e. the log-transformed stationary variance (Khabbazian *et al.* 2016, Bartoszek *et al.* 2017), underlying the evolution of the D, S, and L genes.

In our OU model, we assume that the gene expression vector for D, S, and L genes $e=\left(e_{D},e_{S},e_{L}\right)\in R^{3}$ for a given condition is distributed as multivariate normal (MVN) with mean μ and covariance matrix Σ (Brawand *et al.* 2011), denoted by $e\;∼\;\mathrm{MVN}\left(\mu ,\Sigma \right)$. Therefore, the p=3m-dimensional input expression vector across m conditions is given by

(1) $$x=\left(e_{D1},e_{S1},e_{L1},\ldots ,e_{Dm},e_{Sm},e_{Lm}\right)\in R^{3m},$$

where $e_{jk}$ is the expression measurement for gene $j\in \left\{D,\;S,\;L\right\}$ in condition $k\in \left\{1,2,\ldots ,m\right\}$ of a given deletion event (see Section 2).

Following the approach used by PiXi (Piya *et al.* 2023), we use expression data to predict evolutionary targets and parameters of gene deletions. Given the input feature vector x, we seek to predict the output response y, which for classification is a single qualitative value for the label from either of the K=2 classes “redundant” and “unique”, and for regression is the 3m-dimensional vector of quantitative responses for 3m parameter estimates $\theta_{1}$, $\theta_{2}$, and $\log_{10}\left(\sigma^{2}/(2\alpha )\right)$ in each of the m conditions. For these classification and regression tasks, we follow the approaches of DeGiorgio and Assis (2021) and Piya *et al.* (2023) in constructing three CLOUDe architectures that account for diverse linear and nonlinear relationships between x and y: multi-layer neural network (NN), random forest (RF), and support vector machine (SVM), in addition to a newly implemented extreme gradient boosting architecture (XGB).

### 2.2 Modeling gene expression as an OU process

Following Brawand *et al.* (2011), gene expression $e=\left(e_{D},e_{S},e_{L}\right)\in R^{3}$ in each condition is distributed as multivariate normal (MVN) with mean

(2) $$\mu =\left[\begin{array}{c} \left(1-e^{-\alpha T_{\mathrm{DS}}}\right)\theta_{1}\;+\;e^{-\alpha T_{\mathrm{DS}}}\theta_{2}\\ \left(1-e^{-\alpha T_{\mathrm{DS}}}\right)\theta_{1}\;+\;e^{-\alpha T_{\mathrm{DS}}}\theta_{2}\\ \theta_{2} \end{array}\right]\in R^{3}$$

and covariance matrix

(3) $$\Sigma =\frac{\sigma^{2}}{2\alpha }\left[\begin{array}{ccc} 1 & e^{-2\alpha T_{\mathrm{DS}}} & e^{-2\alpha T_{\mathrm{DSL}}}\\ e^{-2\alpha T_{\mathrm{DS}}} & 1 & e^{-2\alpha T_{\mathrm{DSL}}}\\ e^{-2\alpha T_{\mathrm{DSL}}} & e^{-2\alpha T_{\mathrm{DSL}}} & 1 \end{array}\right]\in R^{3\times 3},$$

denoted by $e\;∼\;\mathrm{MVN}\left(\mu ,\Sigma \right)$. Here, $T_{\mathrm{DSL}}$ denotes the time since the gene duplication event and is scaled to have a value of one, whereas $T_{\mathrm{DS}}$ represents the coalescence time of the D and S gene copies and is drawn uniformly at random within the interval $\left[0,1\right]$. We assume that expression is independent across conditions, but this assumption can be relaxed to account for inter-condition expression covariance (Revell 2008, Revell and Collar 2009, Eastman *et al.* 2011, Clavel *et al.* 2015).

### 2.3 Construction of the CLOUDe NN, XGB, RF, and SVM predictors

We closely followed the procedure outlined by DeGiorgio and Assis (2021) to design a dense feed-forward NN, with the exception of considering two additional hidden layers, i.e. L∈{0,1,2,3,4,5} (Supplementary Tables S5 and S6). Similarly, we used the approach of Piya *et al.* (2023) to construct RF and SVM predictors (Supplementary Table S5). For the construction of the XGB architecture (Supplementary Table S6), we used extreme gradient boosted decision trees with maximum depths D∈{1,2,3,4,5,6} with p=3m input features. Extreme gradient boosting is an ensemble method that combines the results from sequential weak decision trees to produce a stronger final outcome (Chen and Guestrin 2016). As with other implementations of gradient boosting algorithms (Drucker and Cortes 1995), in extreme gradient boosting each decision-tree-like predictor attempts to correct the errors of its predecessor (Chen and Guestrin 2016). This correction is specifically achieved by applying gradient descent, which minimizes the cost when adding new learners (Drucker and Cortes 1995, Chen and Guestrin 2016). Then the final prediction for a given observation is the weighted mean of predictions from each tree, leading to a more precise result (Drucker and Cortes 1995, Chen and Guestrin 2016). For the regression problem, the predictions are the final result, whereas for the classification problem, two probabilities (one for each class) are predicted, and then the observation is classified according to the class with the highest probability. These four machine learning architectures were implemented in R (R Core Team 2021), using Keras (Chollet *et al.* 2017) with a TensorFlow backend (Abadi *et al.* 2016) for the NN, xgboost (Chen and Guestrin 2016) for the XGB, ranger (Wright and Ziegler 2017) for the RF, and liquidSVM (Steinwart and Thomann 2017) for the SVM.

### 2.4 Training and testing the CLOUDe NN, XGB, RF, and SVM predictors on simulated data

As with their construction, we followed DeGiorgio and Assis (2021) and Piya *et al.* (2023) in training and testing the NN, RF, and SVM architectures of CLOUDe, in addition to the newly implemented XGB architecture. We first generated a balanced training dataset with 20 000 observations (10 000 from each class) and an independent balanced test dataset with 2000 observations (1000 from each class). To generate these observations, we assumed m=6 independent conditions, which is the number of tissues in the empirical dataset from *Drosophila* on which we later applied our method (see *Application of CLOUDe to empirical data from Drosophila*), for a total of p=18 input features. For both datasets, parameters $\theta_{1},\theta_{2}$, α, and $\sigma^{2}$ were sampled independently across many orders of magnitude, i.e. $\theta_{1},\theta_{2}\in [0,5]$, $\log_{10}\left(\alpha \right)\in [0,3]$, and $\log_{10}\left(\sigma^{2}\right)\in \left[-2,3\right]$. These specific ranges were chosen to capture the full distributions of potential parameter values, thus aiming to not inflate model performance, as done for PiXi (Piya *et al.* 2023) and CLOUD (DeGiorgio and Assis 2021). Specifically, the range for $\theta_{1}$ and $\theta_{2}$ was matched to that observed in the empirical dataset used in CLOUDe (see *Application of CLOUDe to empirical data from Drosophila*), whereas those for $\log_{10}\left(\alpha \right)$ and $\log_{10}\left(\sigma^{2}\right)$ were matched to wide ranges used in several previous studies (Hansen 1997, Butler and King 2004, Rohlfs *et al.* 2014, Rohlfs and Nielsen 2015, DeGiorgio and Assis 2021, Piya *et al.* 2023). Thus, unless there is knowledge about these parameter ranges in a particular study system, we recommend that the same settings for $\log_{10}\left(\alpha \right)$ and $\log_{10}\left(\sigma^{2}\right)$ be used for other empirical analyses.

In our implemented rejection sampling step, parameters $\theta_{1},\theta_{2}$, α, and $\sigma^{2}$ were repeatedly drawn for each simulated observation until a set of expression values consistent with the empirical values were obtained across all tissues for that observation. Here, the class was determined to be “redundant” when $\theta_{1}=\theta_{2}$ and “unique” when $\theta_{1}\neq \theta_{2}$. We simulated gene expression data $x\in R^{3m}$ under model parameters for a given class, generating 10 000 simulated replicates of parameter values. Then, we followed DeGiorgio and Assis (2021) and Piya *et al.* (2023) to train the NN, RF, and SVM, specifically using different hyperparameter settings for each (Supplementary Table S5). For the NN, we used 5-fold cross-validation to estimate optimal hyperparameters *L*, λ and γ. Whereas *L* is defined as the number of hidden layers in the NN, hyperparameters λ and γ are used to control the degrees of regularization and model sparsity, respectively. We considered six values of *L* ∈{0,1,…,5}, 11 values of γ chosen evenly across [0, 1], and 25 values of $\log_{10}\left(\lambda \right)$ chosen evenly across [-12,-3]. For the RF, we implemented Breiman’s algorithm (Breiman 2001) with T=500 trees, which was chosen to be large enough such that the out-of-bag error plateaued in initial experiments. For the SVM, we used 5-fold cross-validation to estimate hyperparameters γ and *C*. Hyperparameter γ influences the width of the radial basis kernel, whereas *C* is a tuning parameter that defines penalization of observations that violate the margin of the support vectors. We considered seven values of $\log_{10}\left(C\right)$ chosen evenly across [-3, 3], and 11 values of γ chosen evenly across [0.001, 5].

Likewise, the newly implemented XGB architecture was trained using different hyperparameter settings (Supplementary Table S5), and only the model with the lowest cross-validation loss was used for testing. Specifically, we used optimization for up to 500 iterations—with early stopping after 50 rounds without cost minimization—and 5-fold cross-validation to estimate hyperparameters *D* (parameter “max_depth” in xgboost; Chen and Guestrin 2016), γ, λ, and η (Supplementary Table S5). In xgboost (Chen and Guestrin 2016), “max_depth” controls the size of the tree, or the maximum number of decision internal splits in each predictor. Analogous to the NN architecture, λ and γ are used here to control the degrees of regularization and model sparsity, respectively. Thus, interactions of hyperparameters λ and γ in the form of λ(1-γ) and λγ were used as the values for parameters “lambda” and “alpha” in xgboost, respectively. Finally, η (parameter “eta” in xgboost) is the learning rate that acts to shrink the feature weights obtained after each boosting step, making the boosting process more conservative (Chen and Guestrin 2016). We considered six values of *D* ∈{1,2,…,6}, 11 values of γ chosen evenly across [0, 1], 25 values of $\log_{10}\left(\lambda \right)$ chosen evenly across [-12,-3], and four values of η chosen evenly across [0.01, 0.3].

To evaluate whether differences in the sequencing depth of the test or empirical data affects classification performance, we generated new simulated expression values with added noise drawn from a normal distribution with a mean of zero and a standard deviation of 0.001, 0.01, 0.1, or 1. Therefore, a total of four new test sets were generated, each serving as a proxy for expression values derived from transcriptomic data sequenced at different hypothetical depths, with greater noise corresponding to shallower depths. Last, we used the previously trained four models of CLOUDe at optimal settings (Supplementary Table S5) to classify the newly simulated observations. Shapley analysis was performed on the balanced, simulated training dataset using the R package iml (Molnar 2018) and the CLOUDe NN classifier.

### 2.5 Construction of the LRT predictor

After using an OU process to model the expression evolution of deletion events, we used maximum likelihood to estimate their parameters $\theta_{1}$, $\theta_{2}$, α, and $\sigma^{2}$, and then a LRT to classify them as either “redundant” or “unique”. For estimation of evolutionary parameters, we built “unique” and “redundant” models by using general-purpose optimization based on Nelder-Mead (Nelder and Mead 1965) implemented in the “optim” function of the R programming language (R Core Team 2021). We followed Brawand *et al.* (2011) to generate “redundant” and “unique” log-likelihood functions for optimization. Both optimization and log-likelihood functions ran on evolutionary parameters that were drawn independently across many orders of magnitude, with $\theta_{1},\;\theta_{2}\in [0,\;5]$, $\log_{10}\left(\alpha \right)\in [0,\;3]$, $\log_{10}\left(\sigma^{2}\right)\in [-2,\;3]$, and $T_{\mathrm{DS}}\;\in [0,\;1]$, assuming m=6 conditions. As with the CLOUDe architectures, our implemented rejection sampling step assured that parameters $\theta_{1},\theta_{2}$, α, and $\sigma^{2}$ were continuously drawn for each simulated observation until a set of expression values consistent with empirical values were obtained across all conditions for that observation. For classification, we used hypothesis testing in the form of a LRT involving the “redundant” and “unique” models. Specifically, to investigate whether changes in expression optima have occurred, we tested the null hypothesis in which genes in the two lineages share the same optimum ($\theta_{1}=\theta_{2}$, “redundant”) against the alternative hypothesis of different optima ($\theta_{1}\neq \theta_{2}$, “unique”) (Brawand *et al.* 2011). In a LRT, the null hypothesis is nested within the alternative hypothesis (Lewis *et al.* 2011), and the resulting *P*-value is used to assess the probability of each model (Brawand *et al.* 2011), in which *P *<* *.05 provides support for the alternative hypothesis.

### 2.6 Application of CLOUDe to empirical data from *Drosophila*

We applied the best CLOUDe NN models to empirical data consisting of 100 deletion events and their respective expression abundances measured in six tissues of *Drosophila melanogaster* and *Drosophila pseudoobscura* from the Dryad dataset associated with Assis (2019; found at https://doi.org/10.5061/dryad.742564m). To identify these deletions, Assis (2019) performed phylogenetic comparisons across 12 fully sequenced and annotated *Drosophila* species to ascertain orthologous gene families, extracted gene families with sizes of either one or two in both *D.melanogaster* and *D.pseudoobscura*, and used parsimony to infer and polarize deletion events. Of these 100 deletions, 54 occurred in the *D.melanogaster* lineage, and 46 in the *D.pseudoobscura* lineage (Assis 2019). Expression abundances were computed as fragments per kilobase of exon per million fragments mapped (FPKM; Trapnell *et al.* 2013), quantile-normalized, log-transformed, and filtered to remove genes with little or no expression in all tissues (Assis 2019). It is important to note that predictions may be inaccurate if genes are not expressed, and users should therefore ensure that all genes are expressed prior to applying CLOUDe to their data. We applied the trained NN models with 2 hidden layers for the classification problem, and 3 hidden layers for the regression problem, to the 100 deletion events to predict their class as either “redundant” or “unique”, and the 3 parameters $\theta_{1}$, $\theta_{2}$, and $\log_{10}\left(\sigma^{2}/(2\alpha )\right)$.

Of the with 46 L genes in *D.melanogaster*, 11 are associated with lethal phenotypes in FlyBase (Gramates *et al.* 2022). To compare this proportion to the genome-wide proportion, we performed exact binomial tests with the “binom.test” function of the R stats package (R Core Team 2021). Specifically, we set “x” to 11, “n” to 46, and “p” to 0.39 to denote the genome-wide proportion of genes associated with lethal phenotypes in FlyBase (Gramates *et al.* 2022). Of the 11 L genes associated with lethal phenotypes, eight are classified as “unique”. To compare this proportion to the proportion for “redundant” L genes, we set “x” to 8, “n” to 11, and “p” to 0.55 to denote the proportion of deleted “unique” genes.

To evaluate consistency between ranges of empirical and simulated log-transformed expression values, we simulated expression values from the three predicted evolutionary parameters for the empirical data. It is important to note that CLOUDe estimates the log-transformed stationary variance—$\log_{10}\left(\sigma^{2}/(2\alpha )\right)$—rather than $\log_{10}\left(\alpha \right)$ and $\log_{10}\left(\sigma^{2}\right)$ separately for each deletion event. Therefore, there are an infinite number of combinations of α and $\sigma^{2}$ that are compatible with a particular stationary variance. Therefore, we first needed to independently and uniformly at random draw $\log_{10}\left(\alpha \right)\in [0,\;3]$, and then use this value to obtain $\log_{10}\left(\sigma^{2}\right)$ from the predicted stationary variance. We repeated this procedure 200 times for each empirical observation. Because these 200 combinations of α and $\sigma^{2}$ values derive from the same deletion event, we also associated them with the same pair of $\theta_{1}$ and $\theta_{2}$ values predicted for that deletion event. At the end of this process a total of 20 000 derived observations were generated. Then we used CLOUDe to simulate expression data using the $\theta_{1}$, $\theta_{2}$, α, and $\sigma^{2}$ assigned for each observation derived from the empirical dataset, and compared these simulated expression values to the corresponding empirical values.

As a final empirical analysis, we used all “redundant” and “unique” genes in *D.melanogaster* and *D.pseudoobscura* as input for the DAVID Functional Annotation tool (Huang *et al.* 2009, Sherman *et al.* 2022) to perform enrichment analyses of annotated GO terms with default settings. The output represented significant (P<.05) functional enrichments after the Benjamini-Hochberg procedure.

## 3 Results

### 3.1 Prediction performance of CLOUDe

To assess prediction performance of CLOUDe, we trained and tested each of its four architectures on the same independent balanced datasets simulated under “redundant” and “unique” classes (see Section 2). The training set consisted of 20 000 observations (10 000 for each class), and the test set consisted of 2000 observations (1000 for each class). We followed similar training and testing approaches as in DeGiorgio and Assis (2021) and Piya *et al.* (2023), drawing OU parameters $\theta_{1},\theta_{2}$, α, and $\sigma^{2}$ for each dataset independently across many orders of magnitude, i.e. $\theta_{1},\theta_{2}\in [0,5]$, $\log_{10}\left(\alpha \right)\in [0,3]$, and $\log_{10}\left(\sigma^{2}\right)\in \left[-2,3\right]$, so as not to inflate model performance (see Section 2). However, we implemented an additional rejection sampling step in which simulation replicates with expression values that were lower or higher than the respective minimum or maximum expression values in an empirical dataset on which we later applied CLOUDe (see *Analysis of empirical data from Drosophila*) were rejected until a set of expression values consistent with the empirical values was obtained across all conditions. We drew these 4 evolutionary parameters for each of m=6 conditions to match the number of tissues in the empirical dataset, yielding a total of 24 random parameters per simulated replicate. For comparison to our CLOUDe architectures, we also applied a maximum likelihood approach that is classically used in the OU framework (Casella and Berger 2002, Brawand *et al.* 2011, Clavel *et al.* 2015) to the same test data. Specifically, we used maximum likelihood under an OU model to estimate the evolutionary parameters $\theta_{1}$, $\theta_{2}$, α, and $\sigma^{2}\;$under both “redundant” ($\theta_{1}=\theta_{2}$) and “unique” ($\theta_{1}$ and $\theta_{2}$ unconstrained) settings, and used a likelihood ratio test (LRT) to compare the likelihoods of the estimated parameters under these two settings and distinguish between “redundant” and “unique” classes (see Section 2).

**Figure 2.**
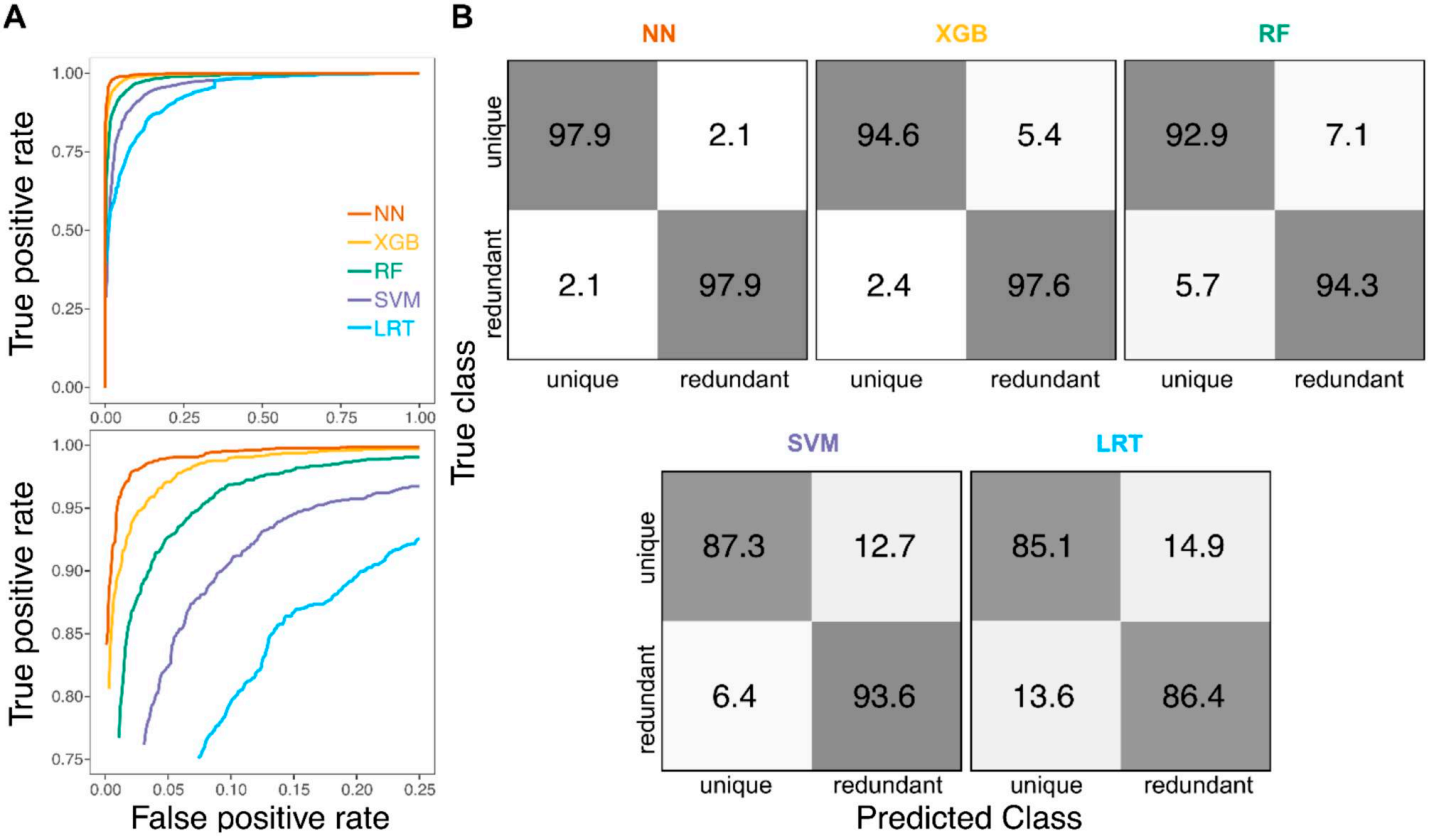
Classification performance of the four CLOUDe architectures and LRT on balanced data simulated under parameters and (A) **log10 (α) ∈** [**0**,] **log10 (σ2) ∈ [—2,3]** Receiver operating characteristic curves evaluating true positive rate (i.e. power) across the full range of false positive rates (top) and zoomed in to show false positive rates ≤25% and true positive rates ≥75% (bottom). (B) Confusion matrices depicting classification rates for the two classes.

We first examined the power and accuracy of each of the four CLOUDe architectures and the LRT in distinguishing between “redundant” and “unique” classes (Fig. 2). Across the wide parameter space considered, classification power is highest with the NN, slightly lower with the XGB, substantially lower with the RF and SVM, and lowest with the LRT (Fig. 2A). Classification accuracy follows a similar trend, with accuracies of 97.90%, 96.10%, 93.60%, 90.45%, and 85.75% for the NN, XGB, RF, SVM, and LRT, respectively (Fig. 2B). Even when instead trained on highly unbalanced “redundant-skewed” or “unique-skewed” datasets (see Section 2), the NN demonstrates higher power and accuracy (96.65% and 96.35%; Supplementary Fig. S1) than the other CLOUDe architectures trained on ideal balanced datasets (Fig. 2). Thus, regardless of the chosen training set, the best overall classification performance is achieved with the NN.

To assess how sequencing depth of the test data affects classification performance, we applied CLOUDe to simulated expression values with varying degrees of added noise (see Section 2), as we expect that lower sequencing depths would provide more uncertainty, and thus elevated noise in measured expression values. We generated a total of four new test sets, each representing a hypothetical degree of noise added to expression values (see Section 2), and applied the CLOUDe classifier to each. We found that CLOUDe is still able to achieve high power in differentiating between “redundant” and “unique” classes for the noise scenarios considered (Supplementary Fig. S2). Specifically, both the NN and XGB retain high power with large amounts of noise, with the NN still the best performer overall. Though these results appear promising, we acknowledge that the performance of CLOUDe, as with any other method, can be hindered by shallow sequencing depth of the transcriptome, and we assume that the expression values used as input to CLOUDe are reliably measured.

As an additional experiment to assess the classification power of CLOUDe, we considered an alternative evolutionary scenario in which the expression optimum for the single-copy gene prior to duplication in the ancestor is $\theta_{0}$, which is permitted to differ from $\theta_{1}$ and $\theta_{2}$. In this scenario, the expression optima of the duplicate genes immediately after duplication in the ancestor are denoted by $\theta_{1}$ and $\theta_{2}$. Following the original scenario considered here, $\theta_{1}$ denotes the expression optima for D and S genes, whereas $\theta_{2}$ denotes the expression optimum for the L gene (Supplementary Fig. S3A). We then generated a new test dataset using this model, and used the previously trained CLOUDe NN, XGB, RF, and SVM models to classify simulated observations. We found that in this alternate scenario, CLOUDe still achieves high power in differentiating between “redundant” and “unique” classes (Supplementary Fig. S3B).

**Figure 3.**
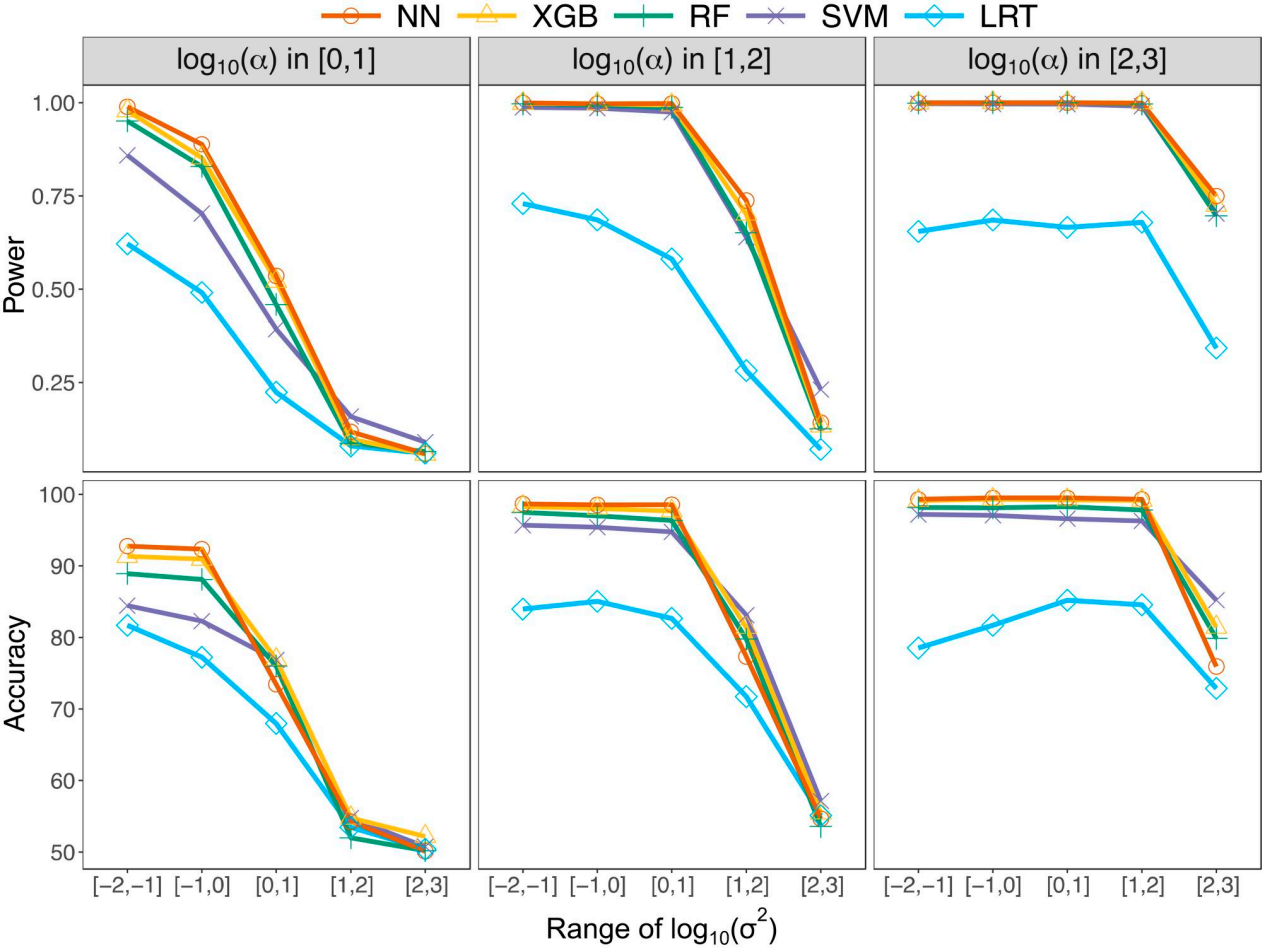
Classification performance of the four CLOUDe architectures and LRT for balanced data simulated under specific parameter ranges for α and $\sigma^{2}$. Top: power curves in which each datapoint represents the true positive rate at a 5% false positive rate for a pair of ranges for α and $\sigma^{2}$. Bottom: accuracy curves in which each datapoint represents the accuracy for a pair of ranges for α and $\sigma^{2}$. For additional ranges of α and $\sigma^{2}$, see Supplementary Fig. S4 and Supplementary Table S1.

Given that CLOUDe retains high classification power even when an alternative evolutionary scenario is considered, for practical purposes we elected to proceed with the original scenario presented in Fig. 1, investigating next how the classification power and accuracy of the four CLOUDe architectures and the LRT vary across smaller regions of the parameter space with combinations of strength of selection (α) and phenotypic drift ($\sigma^{2}$) representing specific evolutionary scenarios (Fig. 3). Consistent with our findings for the broad parameter space (Fig. 2), the four CLOUDe architectures generally show comparable classification power and accuracy in smaller regions of the parameter space, perhaps because drawing test data from a restricted parameter space yields similar values of features across conditions. As in related studies (DeGiorgio and Assis 2021, Piya *et al.* 2023), these methods tend to have highest power and accuracy when selection is strong (large α) or phenotypic drift is weak (small $\sigma^{2}$; Supplementary Fig. S4), and lowest power when selection is weak (small α) or phenotypic drift is strong (large $\sigma^{2}$; Fig. 3 and Supplementary Table S1). Also, consistent with our findings for the broad parameter space (Fig. 2), all four CLOUDe architectures typically have substantially higher power and accuracy than the LRT when the parameter space is restricted. The LRT performs relatively poorly for almost all pairs of ranges for α and $\sigma^{2}$, with low power and accuracy even in the ideal classification scenario with strong selection (large α) and weak drift (small $\sigma^{2}$), when it often misclassifies “unique” observations as “redundant” (Supplementary Fig. S5). A possible explanation for this finding is that, as opposed to the four CLOUDe methods, classification with the LRT is conditional on maximum likelihood estimates of five model parameters ($\theta_{1}$, $\theta_{2}$, α, $\sigma^{2}$, and $T_{\mathrm{DS}}$). Because there are only three sets of input features, these parameters may not be estimated well, resulting in higher misclassification rates with the LRT than with any of the CLOUDe architectures. Overall, CLOUDe demonstrates uniformly high classification power and accuracy across a wide range of evolutionary parameters, regardless of the chosen architecture, in a similar manner as its predecessors (DeGiorgio and Assis 2021, Piya *et al.* 2023).

**Figure 4.**
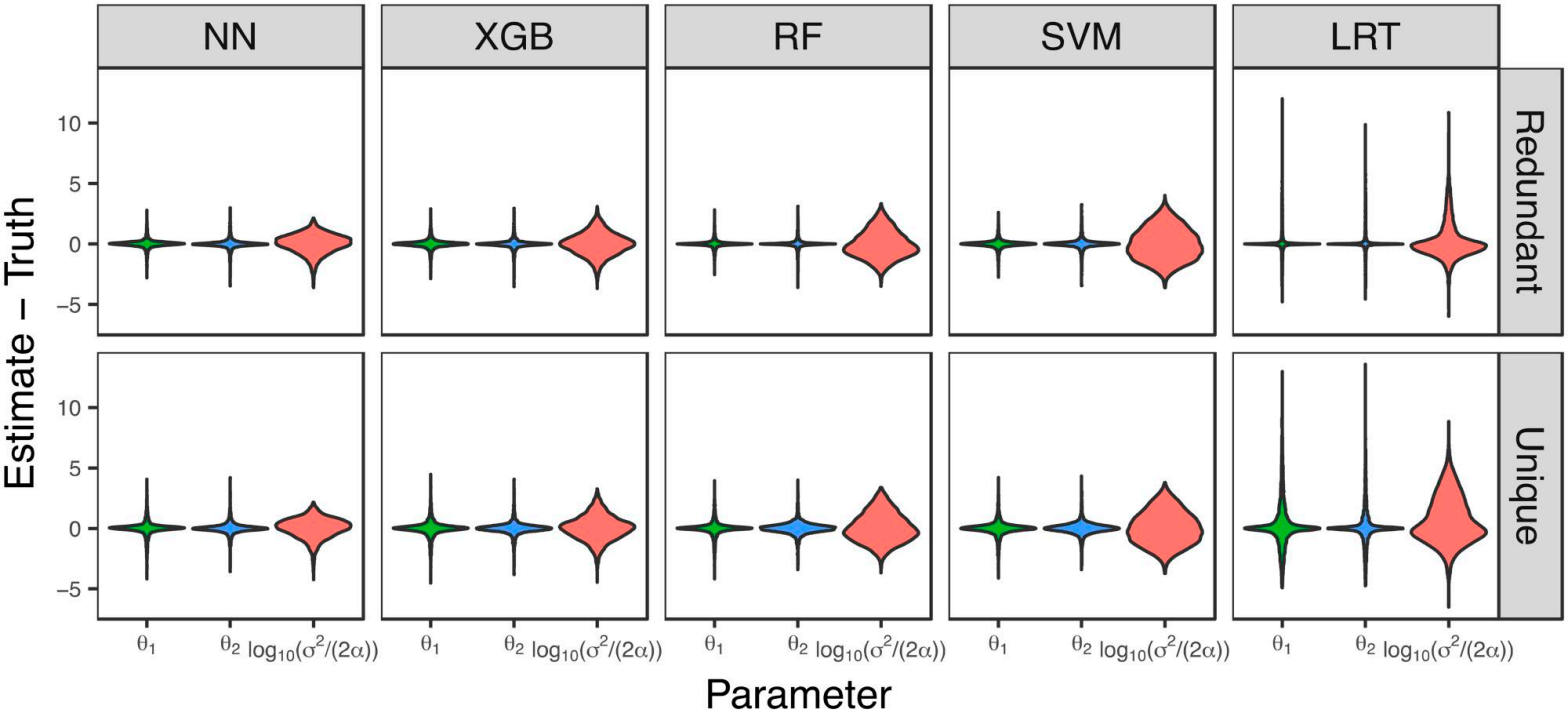
Parameter prediction performance of the four CLOUDe architectures and LRT for data simulated under parameters $\log_{10}\left(\alpha \right)\in [0,3]$ and $\log_{10}\left(\sigma^{2}\right)\in \left[-2,3\right]$. Violin plots display distributions of parameter prediction errors across m=6 conditions.

Last, we assessed the accuracy and precision of each of the four CLOUDe architectures and the LRT in predicting evolutionary parameters $\theta_{1},\theta_{2}$, and $\log_{10}\left(\sigma^{2}/(2\alpha )\right)$ by examining distributions of their prediction errors (Fig. 4). This analysis revealed that parameter predictions of all methods are generally accurate, with errors centered approximately on zero (Supplementary Table S2), mirroring findings from related studies (DeGiorgio and Assis 2021, Piya *et al.* 2023). Also consistent with prior findings (DeGiorgio and Assis 2021, Piya *et al.* 2023), comparisons of distribution widths show that precision is notably higher for $\theta_{1}$ and $\theta_{2}$ than for $\log_{10}\left(\sigma^{2}/(2\alpha \right)$), as well as higher for the “redundant” than for the “unique” class, likely due to the additional degree of freedom in estimating parameters for the “unique” class. Despite these differences, all four CLOUDe architectures display higher precision than the LRT in parameter estimation for both classes, with the NN again outshining the others by also demonstrating the highest precision for estimating $\log_{10}\left(\sigma^{2}/(2\alpha )\right)$.

**Figure 5.**
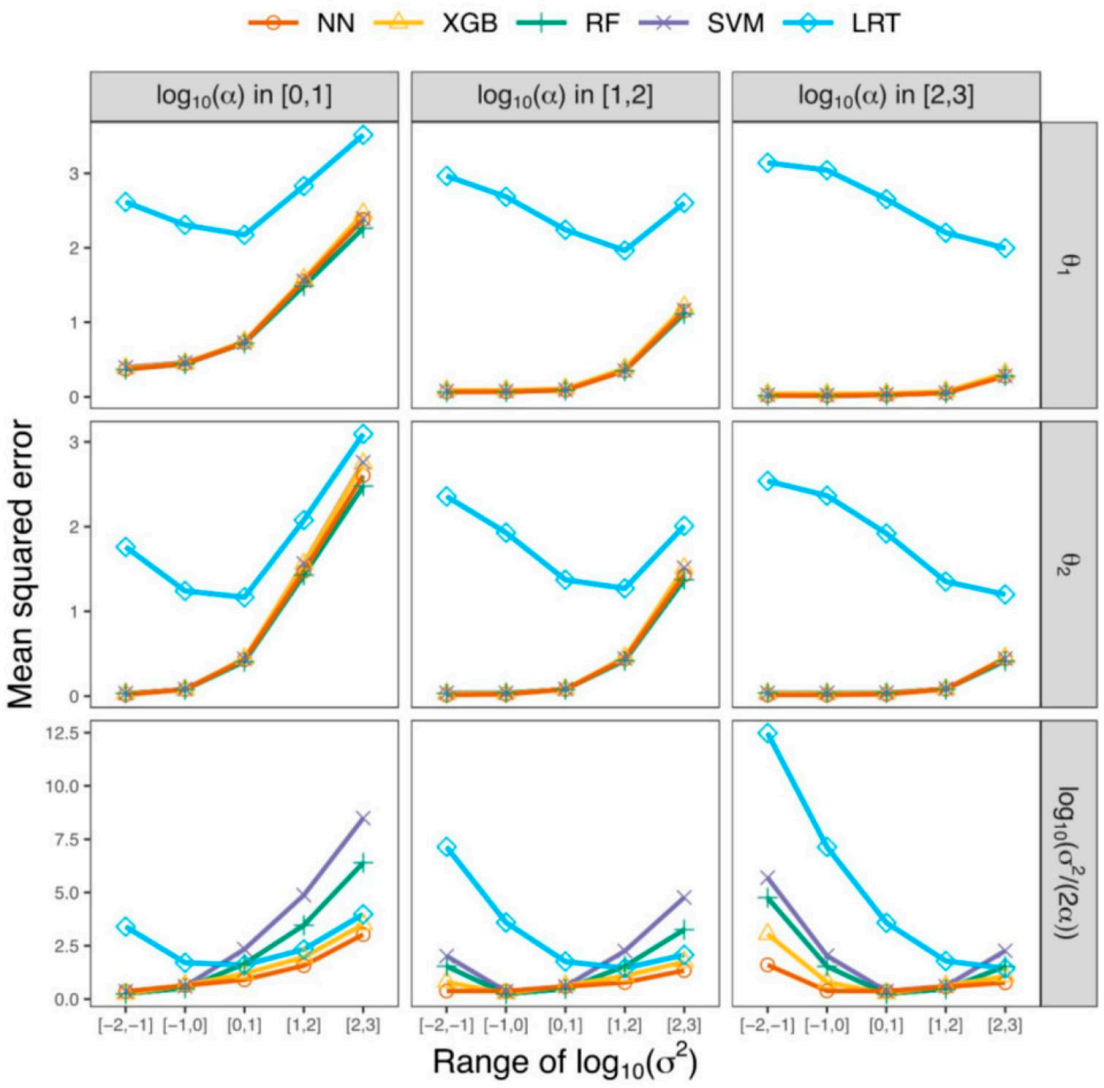
Parameter prediction performance of the four CLOUDe architectures and LRT for data simulated under specific parameter ranges for α and $\sigma^{2}$. Each datapoint represents the mean squared error of a parameter estimate (rows) for each pair of α (columns) and $\sigma^{2}$ (*x*-axes) across m=6 conditions. For additional ranges of α and $\sigma^{2}$, see Supplementary Figs S6 and S7, and Supplementary Table S1.

As with classification, prediction performance of CLOUDe is dependent on values of α and $\sigma^{2}$ (Fig. 5, Supplementary Figs S6 and S7; Supplementary Table S1). However, this dependence differs among the parameter estimates. Specifically, prediction performance for the expression optima $\theta_{1}$ and $\theta_{2}$ tends to be best when selection is strong (large α) and drift is weak (small $\sigma^{2}$), as found in prior studies of related methods (DeGiorgio and Assis 2021, Piya *et al.* 2023). On the other hand, prediction performance for $\log_{10}\left(\sigma^{2}/(2\alpha )\right)\;$is best when drift is slightly weaker than selection. Moreover, though all four CLOUDe architectures demonstrate comparable overall performance in predicting expression optima in most evolutionary scenarios, the NN noticeably outperforms the others in predicting $\log_{10}\left(\sigma^{2}/(2\alpha )\right)$ when drift is strong (large $\sigma^{2}$) or weak (small $\sigma^{2}$). Last, similar to our findings for restricted parameter spaces (Fig. 3), all four CLOUDe architectures typically outperform the LRT by a considerable margin—though this is mitigated for $\log_{10}\left(\sigma^{2}/(2\alpha )\right)$. Overall, the LRT performs relatively poorly for almost all pairs of ranges for α and $\sigma^{2}$, with high error even in the ideal classification scenario with strong selection (large α) and weak drift (small $\sigma^{2}$).

As a final analytical procedure, we conducted Shapley analysis on the NN classifier to investigate the importance of each feature for classification (see Section 2). We found that features associated with the L gene are most important for discriminating between classes (Supplementary Fig. S8). This finding is consistent with how classes are defined in CLOUDe, as only the expression optimum of the L gene is allowed to be different from the expression optima of the D and S genes, ultimately defining the prediction problem for a given observation.

### 3.2 Analysis of empirical data from *Drosophila*

Our simulation analyses demonstrate that CLOUDe has high power and accuracy in predicting evolutionary targets, and high accuracy and precision in predicting evolutionary parameters, of gene deletions, with the best overall performance achieved by its NN architecture. We thus applied the CLOUDe NN to predict evolutionary targets and parameters of gene deletion in *Drosophila* from expression data measured in six tissues (Assis 2019). We specifically analyzed 100 deletion events that occurred in either the *D.melanogaster* or *D.pseudoobscura* lineage after 1. Note that, unlike for the simulated data, the true classes of these 100 gene deletion events are unknown.

Of the 100 deletion events examined, CLOUDe classified 55 as belonging to the “unique” class. Thus, consistent with the results of a previous analysis of these deletions (Assis 2019), CLOUDe predicts that the majority of *Drosophila* duplicate genes possess unique expression profiles prior to deletion. These results are also consistent with findings in many other systems (Hottes *et al.* 2013, Kvitek and Sherlock 2013, Albalat and Cañestro 2016), providing additional support for the “less-is-more” (Olson 1999) rather than the loss of redundancy explanation for gene deletion (Albalat and Cañestro 2016). As expected by our model, “redundant” D, S, and L genes have similar expression across tissues, whereas “unique” L genes have different (and typically lower) expression across tissues than D and S genes (Supplementary Figs S9 and S10). Of the 46 L genes in *D.melanogaster*, 11 (∼24%) are associated with lethal phenotypes (see Section 2), a proportion that is significantly lower than the genome-wide proportion (∼39%; $p=4.81\times 10^{-2},\;\mathrm{exact}\;\mathrm{binomial}\;\mathrm{test}$). Though eight of these 11 L genes (∼73%) belong to the “unique” class, this their divergence (see Section 2), such that there are two gene copies in one species and one gene copy in the other (Assis 2019), as in the scenario depicted in Figure proportion is not significantly different than that for the “redundant” class when we consider that “unique” genes are more often targeted by deletion ($p=3.65\times 10^{-1},\;\mathrm{exact}\;\mathrm{binomial}\;\mathrm{test}$), suggesting that there is no bias toward removing essential genes from either class.

**Figure 6.**
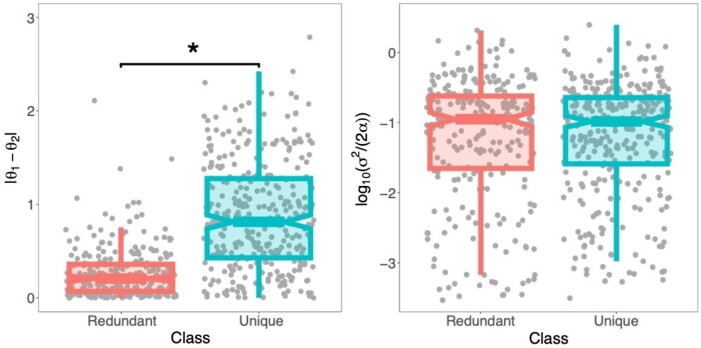
Parameter estimates for the CLOUDe NN applied to empirical data from *Drosophila*. Box plots overlaid onto strip plots showing distributions of absolute differences between predicted expression optima $\theta_{1}$ and $\theta_{2}$, and predicted $\log_{10}\left(\sigma^{2}/(2\alpha )\right)$. Six estimates per deletion event corresponding to the six tissues in the empirical dataset are plotted. *P<.001.

Distributions of absolute differences between predicted $\theta_{1}$ and $\theta_{2}$ (i.e. $\left|\theta_{1}-\theta_{2}|\right)$ and of predicted $\log_{10}\left(\sigma^{2}/(2\alpha )\right)$ differ for the two classes (Fig. 6). In particular, $\left|\theta_{1}-\theta_{2}\right|$ is significantly larger for the “unique” class ($p<2.22\times 10^{-16}$, Mann-Whitney *U* test, see Section 2), consistent with expectations of the OU model underlying CLOUDe. Additionally, predicted $\log_{10}\left(\sigma^{2}/(2\alpha )\right)$ values tend to be negative for both classes ($p=6.96\times 10^{-46}$ for “redundant” and $p=7.30\times 10^{-56}$ for “unique”, Wilcoxon signed-rank tests, see Section 2), perhaps indicating that selection generally plays a larger role than drift in evolution after gene deletion (see Section 4 for other possible reasons). However, distributions of predicted $\log_{10}\left(\sigma^{2}/(2\alpha )\right)$ are not significantly different between “redundant” and “unique” classes, suggesting that the strength of drift relative to that of selection acting on these genes is the same regardless of class. It is important to note that $\log_{10}\left(\sigma^{2}/(2\alpha )\right)$ reflects the stationary variance along the D, S, and L phylogeny (see Fig. 1), suggesting that caution should be taken when interpreting the roles of selection and drift at various evolutionary timepoints involving gene deletion events.

To evaluate consistency between empirical and simulated expression values, we compared empirical expression values to those simulated from parameter predictions obtained from application of CLOUDe to our empirical dataset (see Section 2). We found that distributions of empirical and simulated expression values are similar for D and S genes, but significantly different for L genes ($p=2.70\times 10^{-11}$; Mann-Whitney *U* test), which have larger predicted than empirical values (Supplementary Fig. S11A). One explanation for this observation is that our model allows the expression optimum of the L gene to be different from the expression optima of the D and S genes in the “unique” class, which can result in inflated, but not unexpected, values. However, upon further investigation, we found that this discrepancy may be due to the very low expression of some L genes, as CLOUDe does not predict expression values of zero and rarely predicts expression values close to zero (Supplementary Fig. S11B). Indeed, if we apply a common threshold for expression and remove values with FPKM<1 (i.e. less than ${\log_{10}\left(1+\mathrm{FPKM}\right)=\log}_{10}\left(1+1\right)\approx 0.3$), then the distributions of empirical and simulated expression values are no longer significantly different (Supplementary Fig. S11C).

We next studied functions associated with ancestral pairs of *Drosophila* duplicate genes prior to deletion (S and L genes; Fig. 1) by using DAVID (Huang *et al.* 2009, Sherman *et al.* 2022) to evaluate the enrichment of gene ontology (GO) terms (Ashburner *et al.* 2000, Gene Ontology Consortium *et al.* 2023) in a target gene list against the genome-wide background (see Section 2). We ran DAVID twice, with the target list containing predicted “redundant” genes first, and “unique” genes the second time (see Section 2). Comparisons of statistically significant GO terms between runs revealed distinct functional differences between “redundant” (Supplementary Table S3 and Supplementary Fig. S12A) and “unique” (Supplementary Table S4 and Supplementary Fig. S12B) genes. In particular, “redundant” genes are primarily enriched for functions related to protein processing (biological process), and specifically to acyl transferase activity (molecular function), on the external side of the plasma membrane (cellular component). In contrast, “unique” genes are enriched for functions related to protein deubiquitination (biological process), and specifically to thiol-dependent ubiquitin-specific protease activity (molecular function), in the mitochondrial outer membrane (cellular component).

Last, we performed a case study of the “unique” genes with the largest absolute difference between $\theta_{1}$ and $\theta_{2}$ (i.e. $\left|\theta_{1}-\theta_{2}|\right)$ and the highest magnitude negative log-transformed stationary variance, as such genes display the greatest evidence of uniqueness. These genes represent a scenario in which there was a deletion in the *D.pseudoobscura* lineage, such that the *D.melanogaster* lineage contains the ancestral pair of “unique” duplicate genes *Ran* (*CG1404*, S) and *Ran-like* (*CG7815*, L). In this case, *Ran* is the parent gene that gave rise to a duplicate gene copy *Ran-like* (Tracy *et al.* 2010, Larracuente and Presgraves 2012), which was then deleted in the *D.pseudoobscura* lineage. *Ran* is broadly expressed across all tissues analyzed here and is most highly expressed in ovary, whereas *Ran-like* is tissue-specific and primarily expressed in testis (Kunte 2009, Gramates *et al.* 2022). This case is therefore an example of the long-standing “out of the testis” hypothesis for the origin of genes created by gene duplication (Kaessmann 2010), as well as of the recent “into the ovary” hypothesis, which posits that gene deletion preferentially removes genes that are not highly expressed in ovary, perhaps promoting adaptation by salvaging genes that contribute to the evolution of female reproductive phenotypes (Assis 2019). Indeed, *Ran* is a biologically important gene (Tracy *et al.* 2010, Boudhraa *et al.* 2020, Mirsalehi *et al.* 2021, Gramates *et al.* 2022) with many associated lethal phenotypes, in contrast to no lethal phenotypes observed for *Ran-like* (Gramates *et al.* 2022). Moreover, overexpression of *Ran* are associated with numerous forms of cancers, including ovarian and breast carcinomas (Boudhraa *et al.* 2020). On the other hand, disruptions in the expression of *Ran-like* causes spermatid disfunction and other germline conflicts during spermatogenesis (Kunte 2009, Tracy *et al.* 2010, Larracuente and Presgraves 2012). These conflicts may explain its deletion in the *D.pseudoobscura* lineage, perhaps representing an interesting avenue of future research.

## 4 Discussion

CLOUDe represents the first model-based machine learning framework tailored to the problem of predicting evolutionary targets and parameters of gene deletion from expression data. Specifically, CLOUDe uses an OU model overlaid by NN, XGB, RF, and SVM architectures for predicting whether the targets of gene deletion are “redundant” or “unique”, as well as their expression optima and relative roles of selection and drift in their evolution. Applications of CLOUDe to simulated data demonstrate innately high power and accuracy in differentiating between “redundant” and “unique” genes (Figs 2 and 3), as well as high accuracy and precision in estimating their evolutionary parameters (Figs 4 and 5), regardless of the machine learning architecture used. These analyses also reveal the NN as the globally best performer in predicting both evolutionary targets and parameters of gene deletion. Though they do not exhibit the best performance in our study, the XGB, RF, and SVM architectures of CLOUDe can be of great value in other settings. Specifically, XGB and RF may be ideal when expression data are unavailable for some conditions or genes, as these methods are able to naturally handle missing data (Drucker and Cortes 1995, Breiman 2001, Hastie *et al.* 2009, Chen and Guestrin 2016). The SVM architecture, on the other hand, may be advantageous when there are expression data for one or few conditions, as it can increase dimensionality (Schölkopf *et al.* 2001, Chapelle *et al.* 2006). Therefore, the inclusion of these four machine learning architectures in CLOUDe promotes flexibility in its usage. Additionally, though expression data for multiple of the same conditions in three or more species are currently scarce, future extensions of the CLOUDe framework to more than two species may improve its prediction performance.

Our application of the CLOUDe NN to empirical data from *Drosophila* reveals that deletion often targets genes with unique expression profiles, supporting the hypothesis that gene deletion is not simply an evolutionary mechanism for ridding the genome of redundancy (Olson 1999, Hottes *et al.* 2013, Kvitek and Sherlock 2013, Albalat and Cañestro 2016, Assis 2019). Moreover, predicted expression optima are generally consistent with theoretical expectations for each class (Fig. 6), and predicted log-transformed stationary variances are typically negative for both classes (Fig. 6), implying that selection plays a larger role in the evolution of deleted genes. However, one has to consider that here the log-transformed stationary variance is generally expected to be negative for two reasons: the magnitudes of selection scenarios considered in relation to drift (i.e. stationary variance is proportional to the ratio of $\sigma^{2}$ and α), and the fact that $\log_{10}(\alpha )$ is always non-negative whereas $\log_{10}(\sigma^{2})$ is allowed to be negative when drawing parameters for our simulations. Moreover, our investigation of empirical expression values showed that most “unique” L genes in *Drosophila* are primarily expressed in testis and accessory gland tissues. Thus, many such cases possibly represent examples of the long-standing “out of the testis” hypothesis for the origin of genes created by gene duplication (Kaessmann 2010), as in our case study. Further, functional enrichment analyses of these empirical data show that “redundant” genes are often involved in protein processing activities on the external side of the plasma membrane, whereas “unique” genes are often associated with protein deubiquitination in the mitochondrial outer membrane, suggesting that deletion targets distinct functions when removing “redundant” versus “unique” genes from the genome. Together, these findings support the reliability of CLOUDe predictions.

Last, we wish to highlight that the joint application of CLOUD and CLOUDe can detail the pathway that ultimately leads to the loss of unique genes. For example, a previous application of CLOUD to empirical data from *Drosophila* showed that most duplicate genes rapidly acquire unique expression profiles (DeGiorgio and Assis 2021). Thus, it is not surprising that our application of the CLOUDe NN to deleted genes from the same species indicates that most targets of gene deletion possess unique expression profiles. Further, CLOUD and CLOUDe both predict classes from gene expression, which is widely regarded as an ideal proxy for function, as divergent expression profiles correlate with protein-coding gene sequence divergence (Nuzhdin *et al.* 2004, Subramanian and Kumar 2004, Lemos *et al.* 2005, Hunt *et al.* 2013, Assis and Kondrashov 2014, Jiang and Assis 2017, Mähler *et al.* 2017, Assis 2019) and other functional metrics (Ge *et al.* 2001, Zhou *et al.* 2002, Bhardwaj and Lu 2005, French and Pavlidis 2011). Indeed, our functional enrichment analyses uncovered distinct functions in “redundant” and “unique” genes targeted by deletion in *Drosophila*. Our case study of a pair of “unique” genes also provides support for their unique functions, as these genes are highly expressed in opposite sex tissues (Chippindale *et al.* 2001, Kunte 2009, Patten and Haig 2009, Tracy *et al.* 2010, Domingues 2014). Hence, this example demonstrates how researchers with expression data from duplication and deletion events can combine the output of CLOUD and CLOUDe to shed light on functional outcomes of gene turnover in a biological system of interest.

## Supplementary Material

vbae002_Supplementary_Data

## Data Availability

The data underlying this article are available in GitHub, at https://github.com/anddssan/CLOUDe.
