# Supplementary material for: Predicting evolutionary targets and parameters of gene deletion from expression data
Source: Bioinform Adv. 2024 Jan 17;4(1):vbae002. doi: 10.1093/bioadv/vbae002 (PMC10812876; doi:10.1093/bioadv/vbae002)
Supplement: vbae002_Supplementary_Data [file vbae002_supplementary_data.pdf]

Supplementary Materials for

Predicting evolutionary targets and parameters of gene deletion from expression data

Andre Luiz Campelo dos Santos, Michael DeGiorgio, Raquel Assis

This file includes:

Figures S1 to S12
Tables S1 to S6

Figure S1 | Classification performance of the CLOUDe NN trained on unbalanced "redundant-skewed" and "unique-slewed" datasets. The "redundant-skewed" training set consisted of 16,000 observations of the "redundant" class and 4,000 observations of the "unique" class, whereas the "unique-skewed" training set consisted of 4,000 observations of the "redundant" class and 16,000 observations of the "unique" class. **(A)** Receiver operating characteristic curves zoomed in to show false positive rates <= 25% and true positive rates >= 75%. **(B)** Confusion matrices depicting classification rates for the two classes.

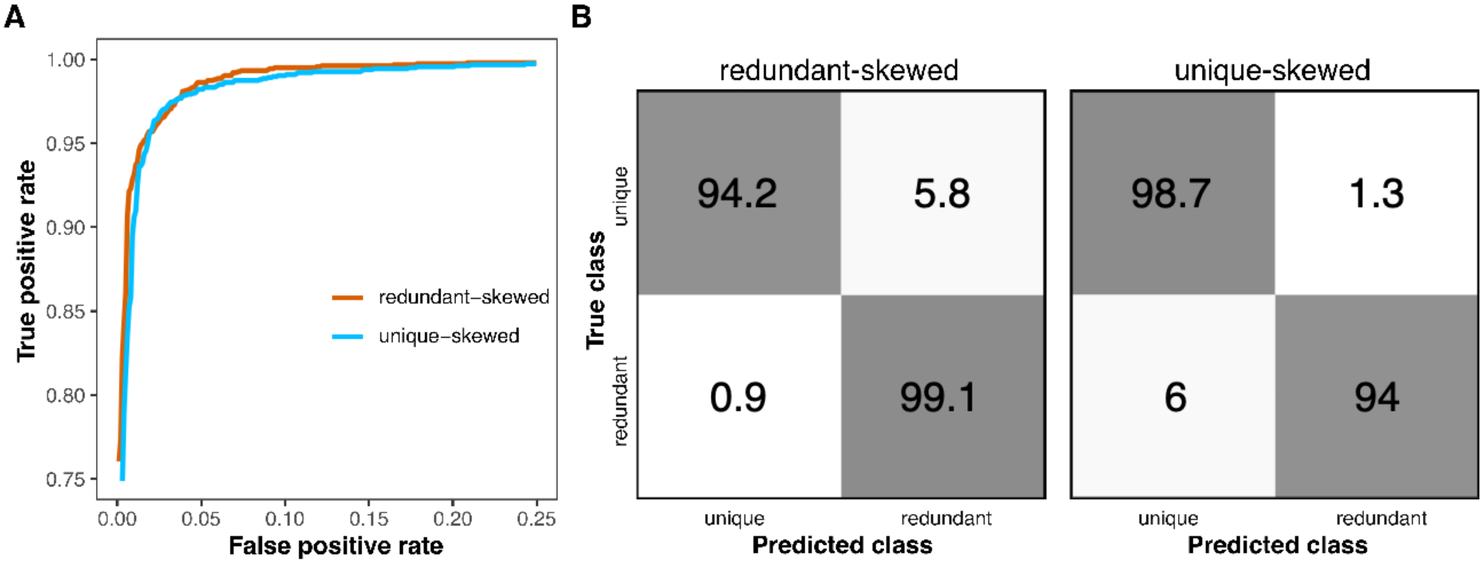

Figure S2 | Classification power of CLOUDe when varying degrees of Gaussian noise are added to simulated test data. Receiver operating characteristic curves zoomed in to show false positive rates <= 25% and true positive rates >= 75%. Given that expression values are log-transformed here, additions of noise with standard deviation (sd) equal to 1 effectively represents the addition of error of different orders of magnitude for the raw non-log-transformed expression values. Noise with mean zero and sd $\in \{0.001, 0.01, 0.1, 1\}$ was added to simulated expression values for D, S, and L genes across the $m = 6$ conditions. Rejection sampling was applied, and final expression values (with added noise) that were higher than the maximum empirical expression value were rejected.

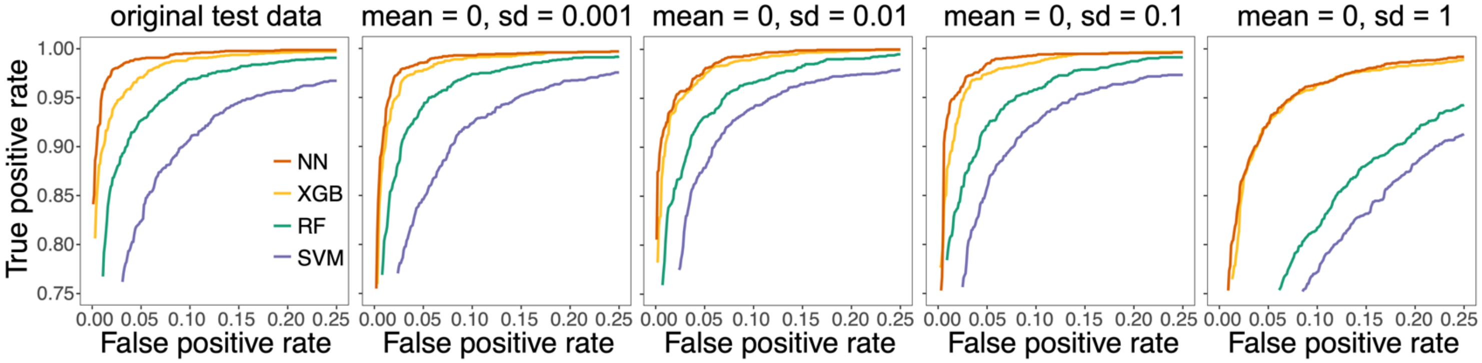

Figure S3 | Classification power of CLOUDe when an alternative evolutionary scenario is considered. (A) In this scenario, the expression optima of the duplicate genes (θ_1 and θ_2) diverged from that of the ancestral gene (θ_0). **(B)** Receiver operating characteristic curves across the full range of false positive rates (left) and zoomed in to show false positive rates <= 25% and true positive rates >= 75% (right).

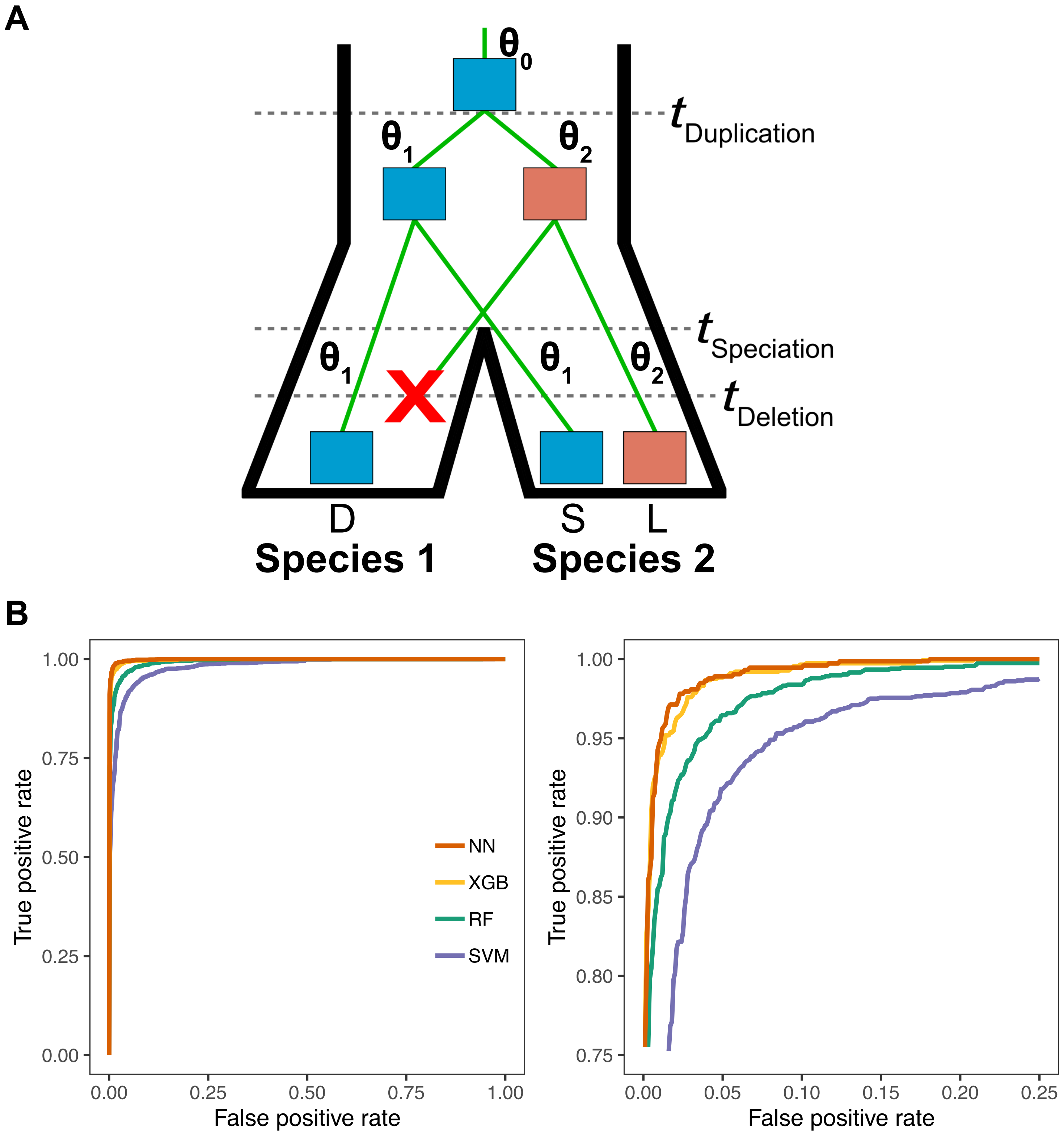

Figure S4 | Classification performance of the four optimal models of CLOUDe (Table S5) applied to balanced test data simulated under ranges for $\alpha \in [0, 10]$ and $\sigma^2 \in [-10, -2]$. **(A)** Power curves zoomed in at power >= 75% in which each datapoint represents the true positive rate at a 5% false positive rate for a pair of ranges for α and σ^2. **(B)** Accuracy curves zoomed in at accuracy >= 82% in which each datapoint represents the accuracy for a pair of ranges for α and σ^2.

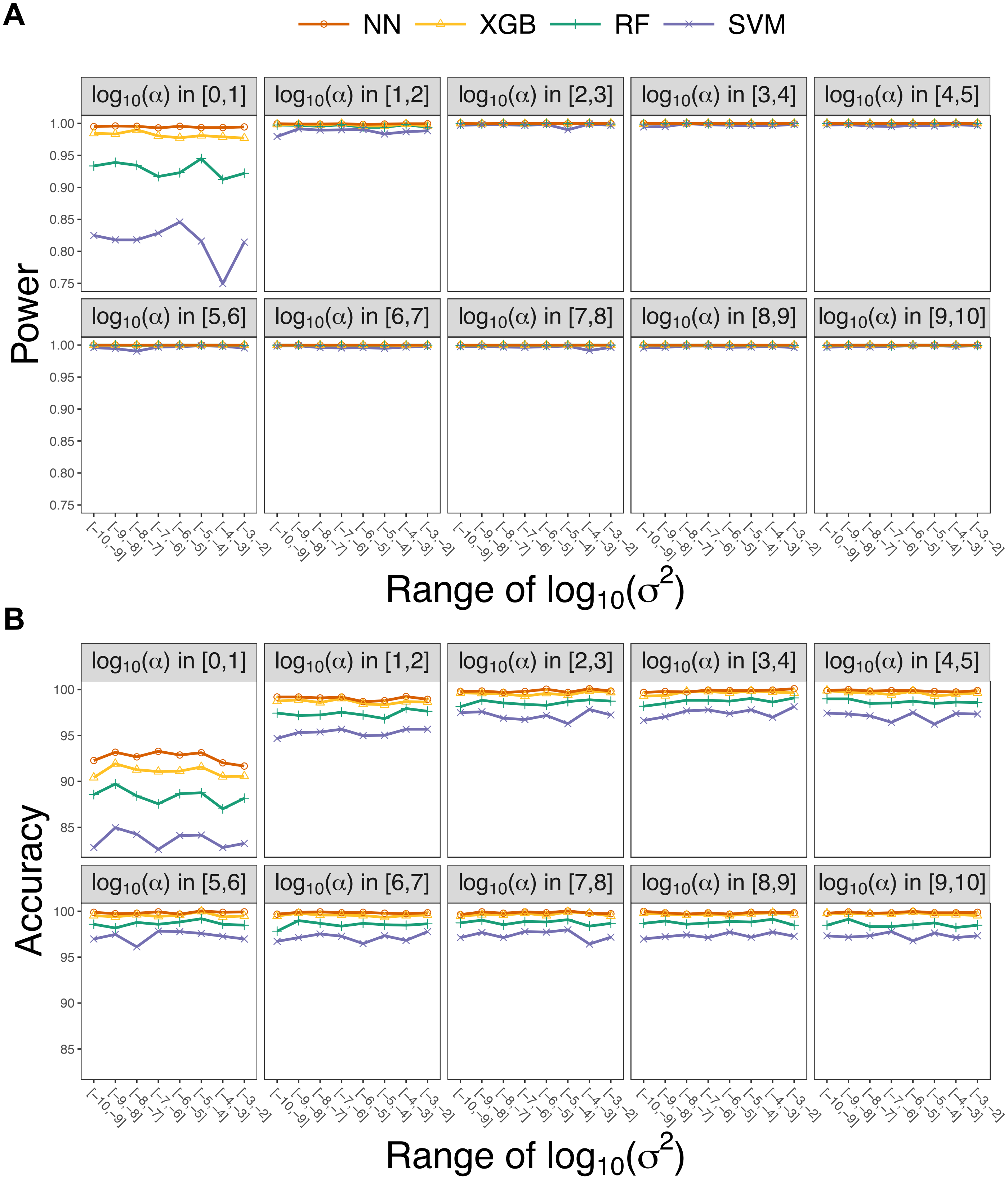

Figure S5 | Confusion matrices depicting classification rates of the LRT for balanced data simulated under specific parameter ranges for α and σ^2. This method is skewed toward predicting the “redundant” class when α is large and σ^2 is small.

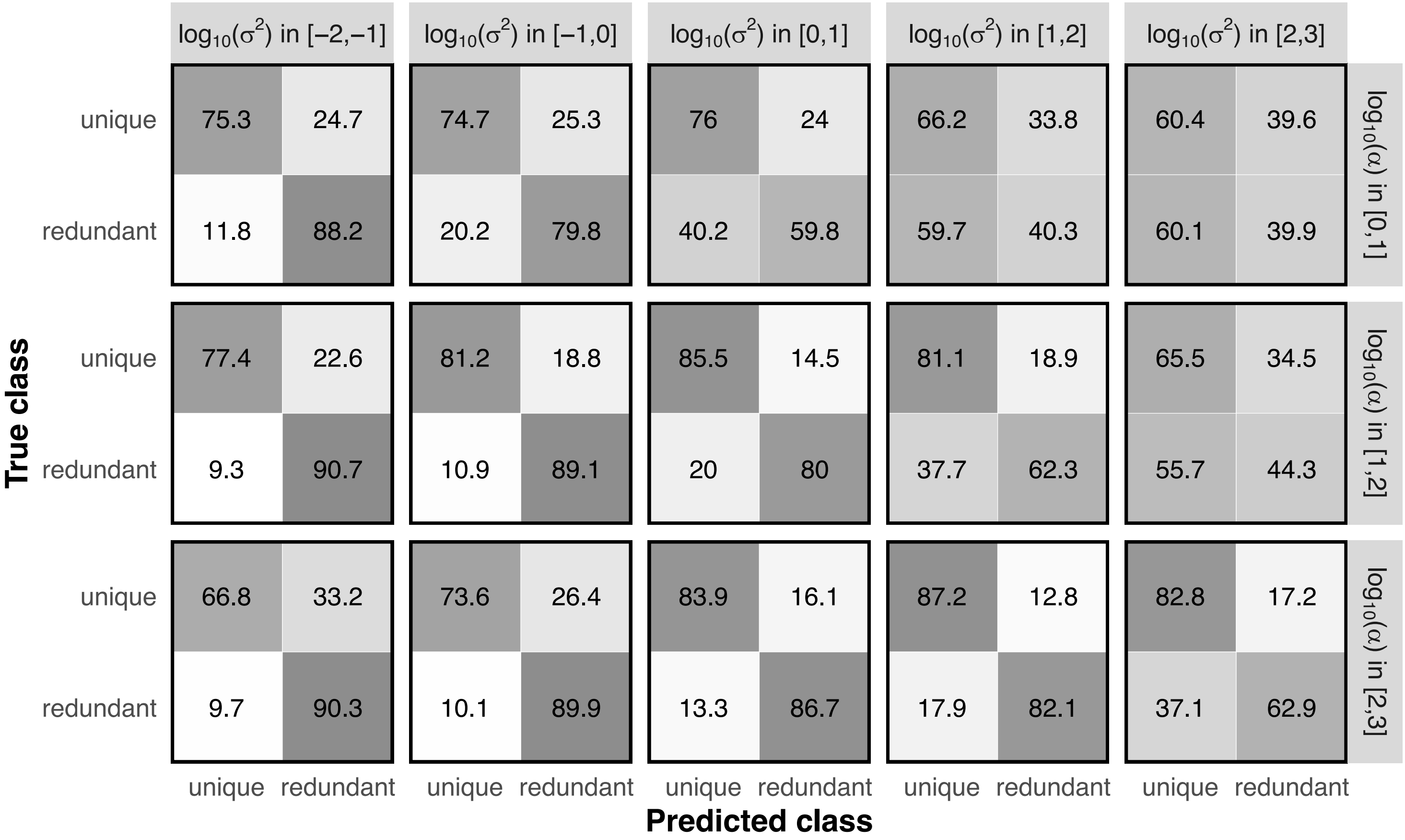

Figure S6 | Parameter prediction performance of the four optimal models of CLOUDe (Table S5) applied to data simulated under ranges for $\alpha \in [0, 10]$ and $\sigma^2 \in [-10, -6]$. Each datapoint represents the mean squared error of a parameter estimate (row) for each pair of α (column) and σ^2 (x-axis) across $m = 6$ conditions.

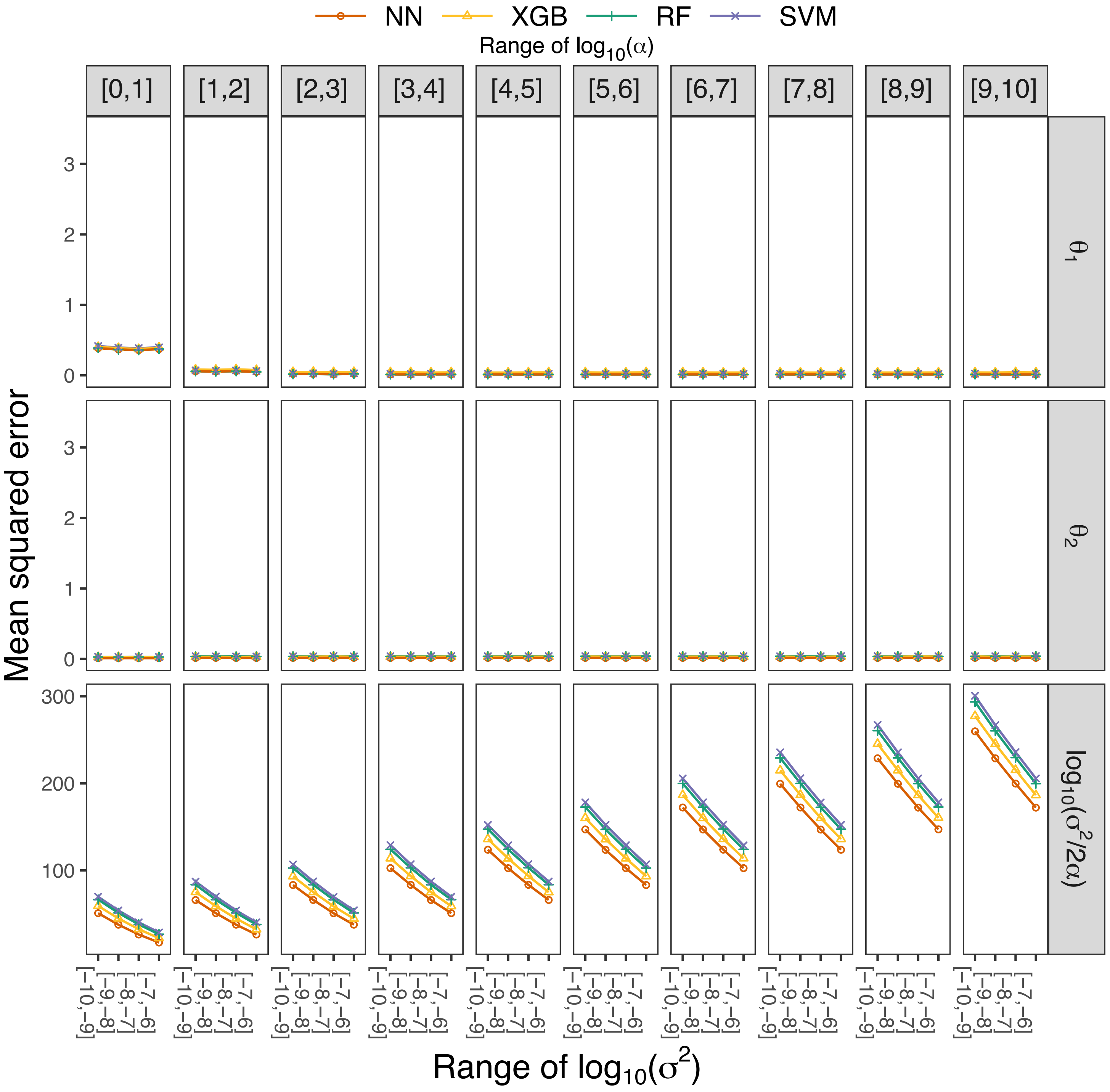

Figure S7 | Parameter prediction performance of the four optimal models of CLOUDe (Table S5) applied to data simulated under ranges for $\alpha \in [0, 10]$ and $\sigma^2 \in [-6, -2]$. Each datapoint represents the mean squared error of a parameter estimate (row) for each pair of α (column) and σ^2 (x-axis) across $m = 6$ conditions.

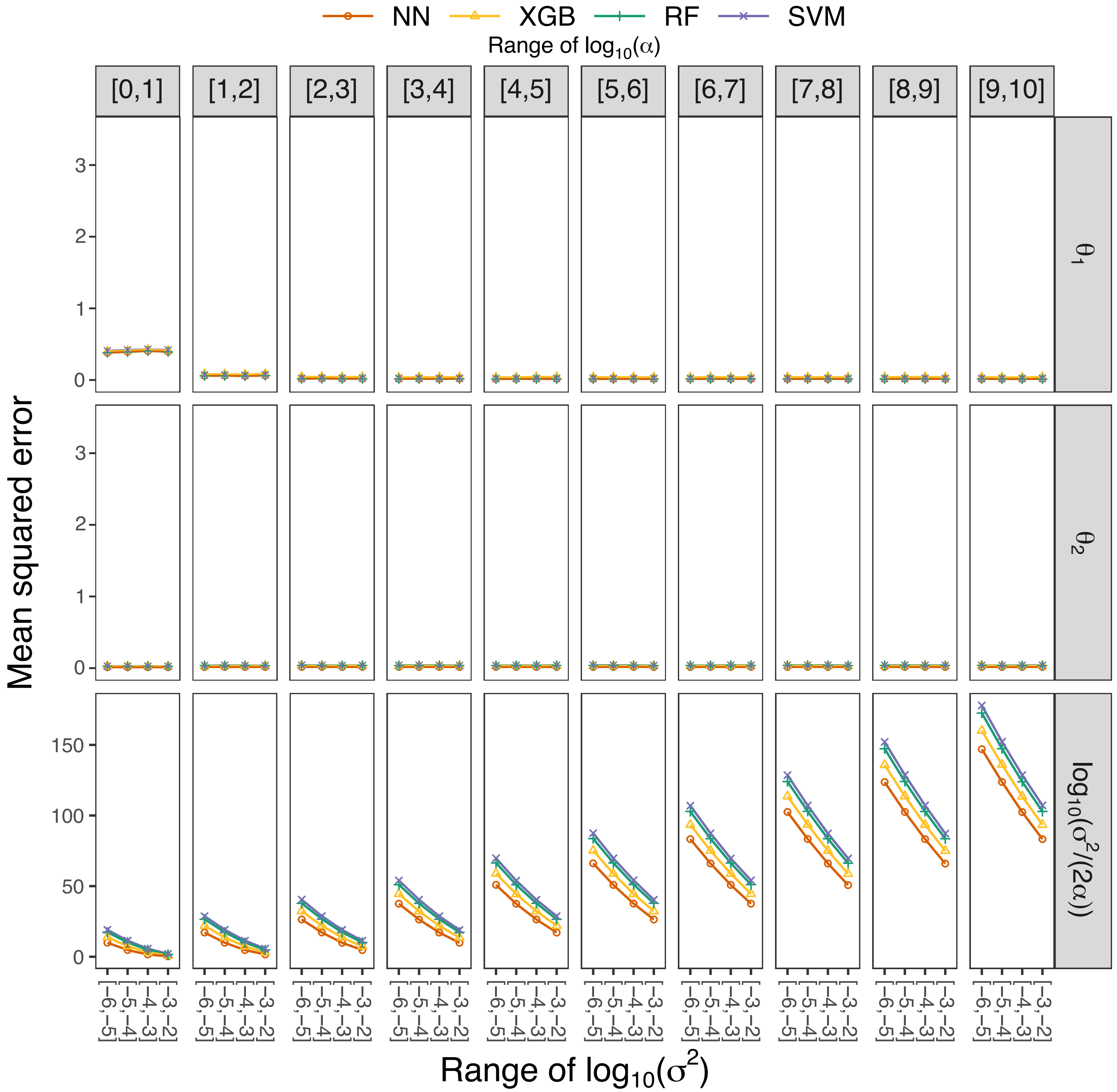

Figure S8 | Shapley analysis of the NN classifier on the simulated training dataset. Points represent Shapley importance values for each feature.

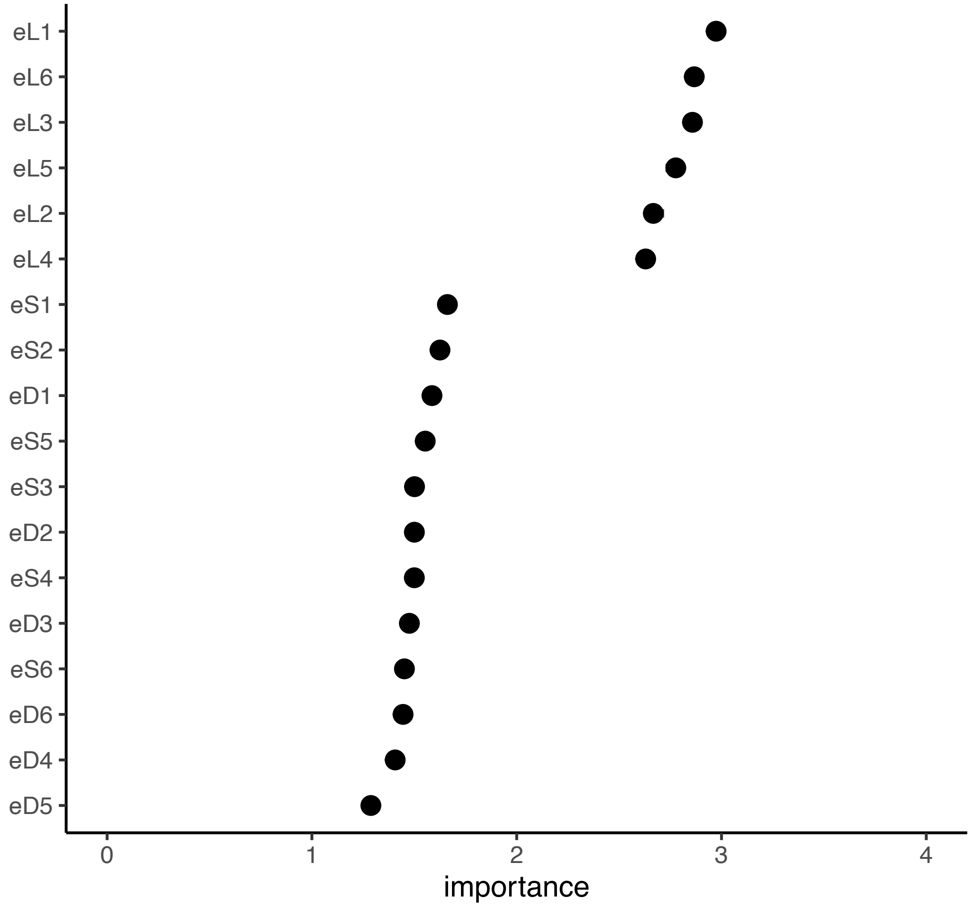

Figure S9 | Gene expression profiles for 100 *Drosophila* deletions, as classified by the CLOUDe NN. Heatmap generated from log-transformed expression values of D, S, and L genes for each deletion event. Labels eX1, eX2, eX3, eX4, eX5, and eX6, represent X = D, S, or L gene expression in the carcass, female head, ovary, male head, testis, and accessory gland, respectively. Rows represent triplets of D, S, and L genes for each of the 100 deletion events.

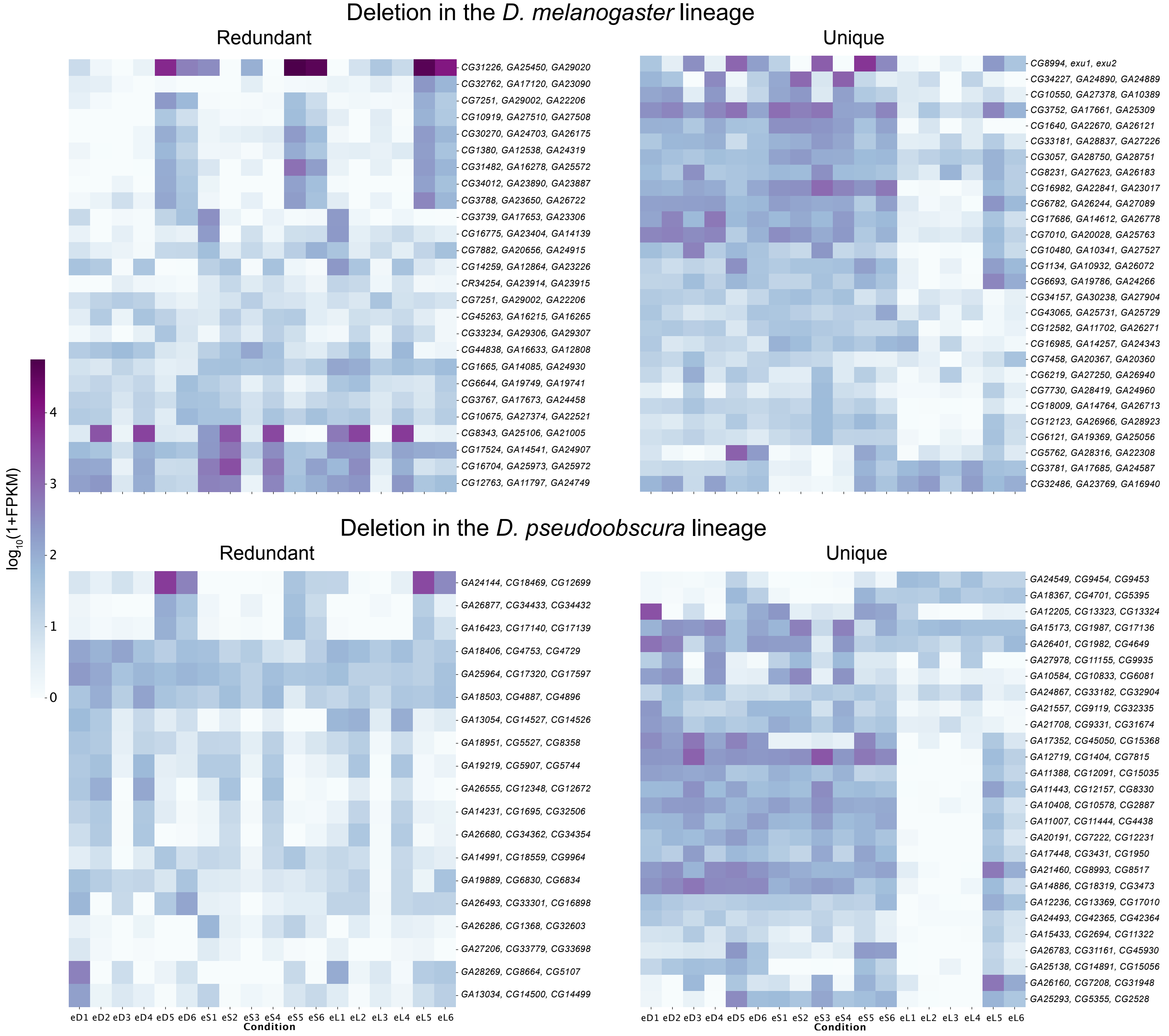

Figure S10 | Distributions of gene expression values for 100 *Drosophila* deletions, as classified by the CLOUDe NN. Log-transformed expression values of D, S and L genes are plotted across $m = 6$ conditions.

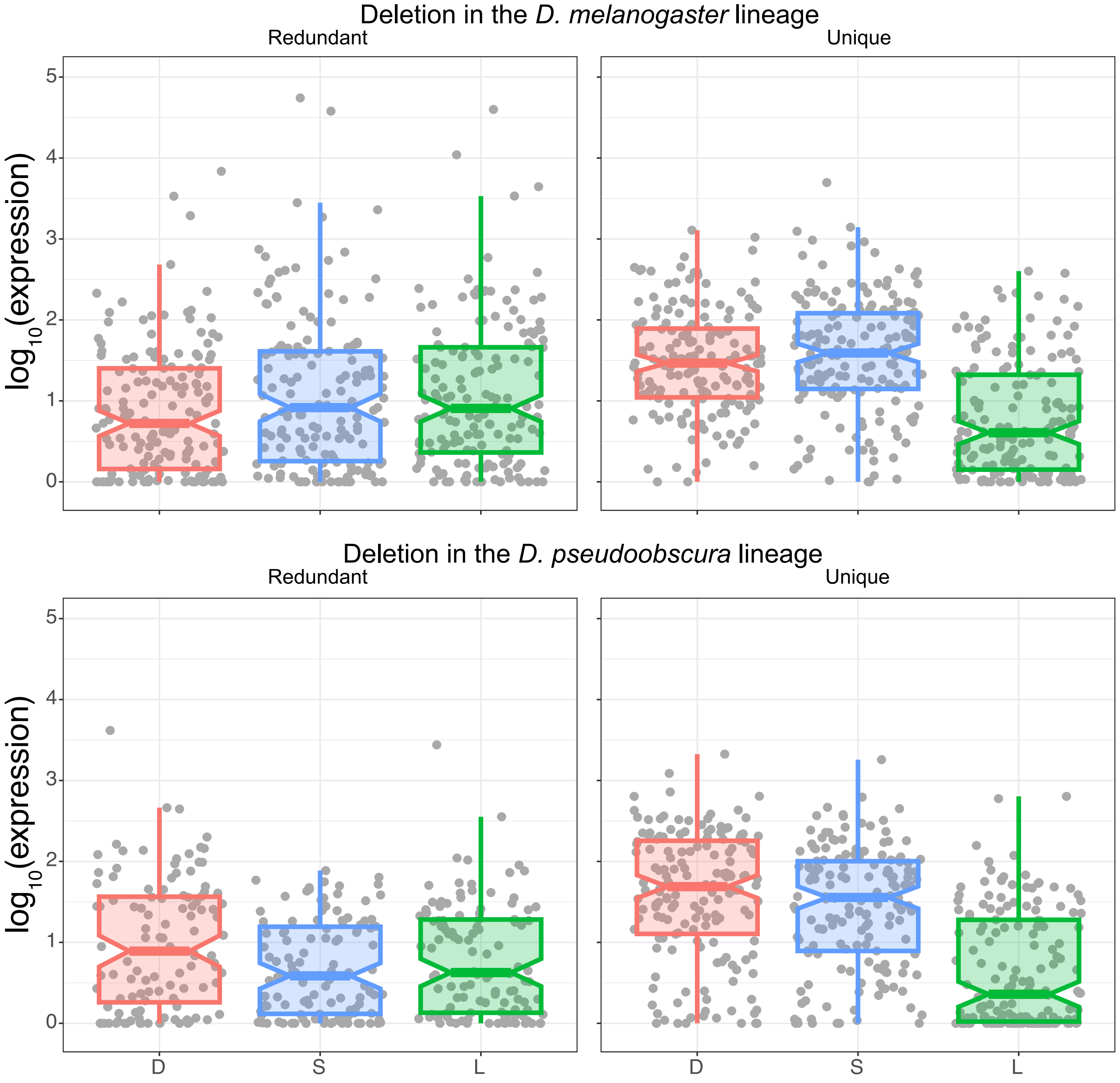

Figure S11 | Distributions of empirical expression values and those simulated from predicted parameters for 100 *Drosophila* deletions. (A) Box plots comparing distributions for D, S, and L genes. **(B)** Frequency polygons comparing distributions for D, S, and L genes in "redundant" and "unique" classes. **(C)** Box plots comparing distributions for D, S, and L genes after removing expression values smaller than FPKM = 1 (i.e., $\log_{10}(\text{FPKM} + 1) = \log_{10}(1 + 1) \approx 0.3$). Log-transformed expression values of D, S, and L genes are plotted across $m = 6$ conditions. "*" indicates $p < 0.001$.

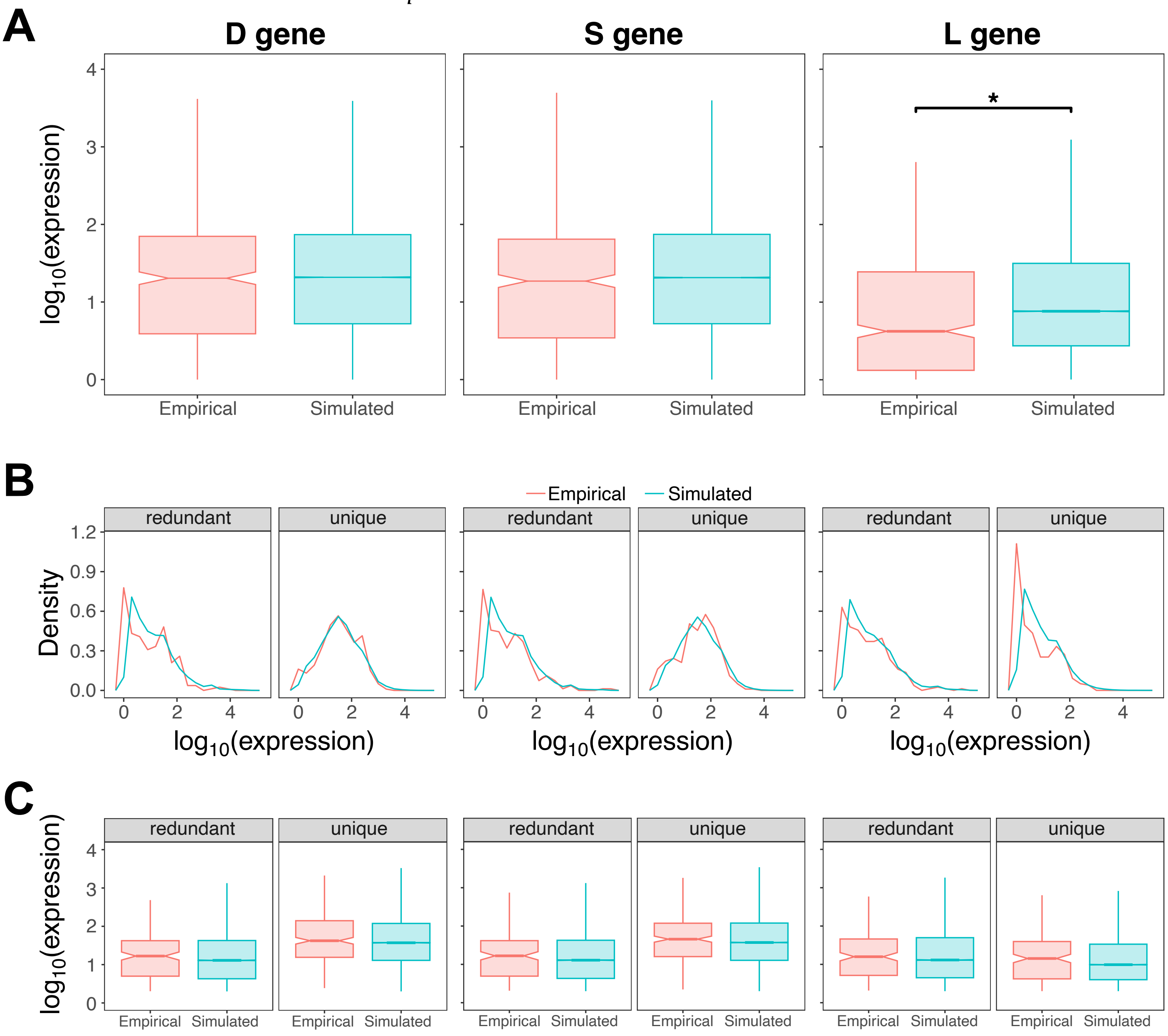

Figure S12 | GO plots depicting statistically significant results of DAVID analyses for the 46 cases in which a deletion occurred in the *D. pseudoobscura* lineage. Results are shown for **(A)** “redundant” and **(B)** “unique” genes, as classified by the CLOUDe NN. The p-value threshold is set to $-\log_{10}(0.05) \approx 1.3$ after Benjamini-Hochberg procedure. Colors of circles reflect GO term categories, whereas their sizes are proportional to the number of genes associated with each GO term category (see Tables S3 and S4).

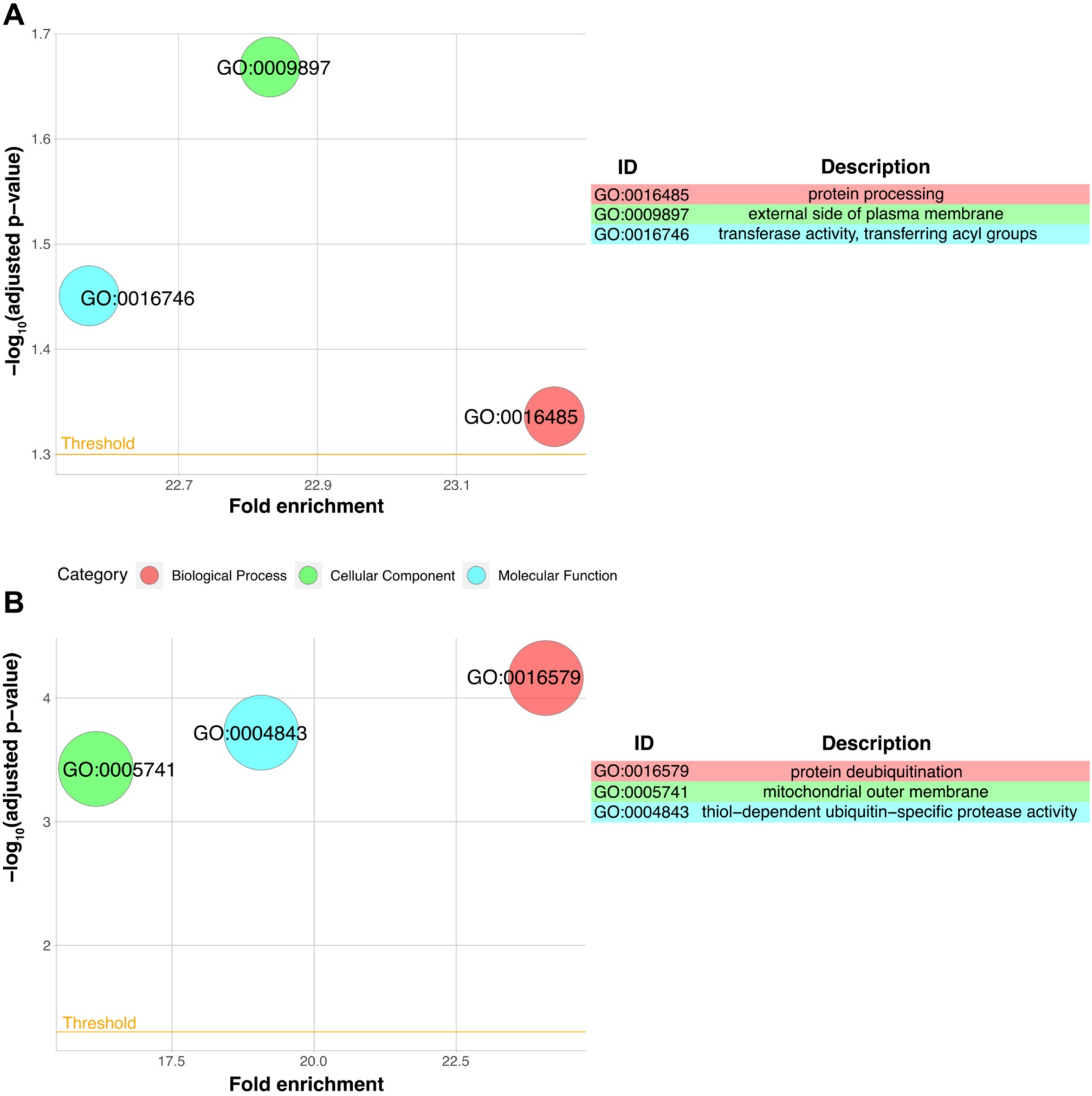

Table S1 | Performance of the four optimal models of CLOUDe (Table S5) applied to data simulated under ranges for $\alpha \in [3, 10]$ and $\sigma^2 \in [3, 10]$. Due to the implemented rejection sampling step for data generation, and the ranges of log-transformed expression values in the empirical data, the Ornstein-Uhlenbeck model of CLOUDe is conservative in finding acceptable simulated expression values for many combinations of $\log_{10}(\alpha) \in [3,10]$ and $\log_{10}(\sigma^2) \in [3,10]$, effectively failing to generate simulated data when $\log_{10}(\sigma^2)$ is three or more orders of magnitude higher than $\log_{10}(\alpha)$. Because it is not possible to explore the entire grid of values for $\log_{10}(\alpha)$ and $\log_{10}(\sigma^2)$, here we present only results for the combinations from which data were generated. Power represents the true positive rate at a 5% false positive rate. MSE_1, MSE_2, and MSE_{sv} denote mean squared errors in predicting θ_1, θ_2, and the log-transformed stationary variance, respectively.

**Spaces**	**NN**					**XGB**					**RF**					**SVM**				
$\log_{10}(\alpha)$; $\log_{10}(\sigma^2)$	**Power**	**Accuracy**	**MSE_1**	**MSE_2**	**MSE_{sv}**	**Power**	**Accuracy**	**MSE_1**	**MSE_2**	**MSE_{sv}**	**Power**	**Accuracy**	**MSE_1**	**MSE_2**	**MSE_{sv}**	**Power**	**Accuracy**	**MSE_1**	**MSE_2**	**MSE_{sv}**
[3,4] ; [3,4]	0.770	76.600	0.282	0.443	0.761	0.743	81.650	0.325	0.458	1.062	0.695	79.550	0.284	0.416	1.524	0.656	84.750	0.286	0.449	2.275
[3,4] ; [4,5]	0.169	55.350	1.099	1.429	11.278	0.157	56.950	1.164	1.524	11.425	0.124	53.800	1.066	1.374	6.308	0.134	59.850	2.106	2.345	4.191
[3,4] ; [5,6]	0.055	49.850	2.267	2.621	13.193	0.050	50.850	2.333	2.734	13.418	0.048	50.100	2.146	2.500	7.389	0.055	51.000	2.534	2.576	4.386
[4,5] ; [3,4]	0.997	99.150	0.048	0.085	0.567	0.999	98.700	0.069	0.086	0.616	0.996	98.050	0.047	0.085	0.471	0.989	96.450	0.054	0.090	0.610
[4,5] ; [4,5]	0.699	76.700	0.276	0.423	0.747	0.722	81.650	0.313	0.440	1.044	0.681	80.400	0.277	0.402	1.500	0.656	85.000	0.278	0.428	2.262
[4,5] ; [5,6]	0.156	55.700	1.087	1.441	18.885	0.165	58.200	1.157	1.537	19.031	0.152	54.550	1.055	1.385	12.175	0.121	58.600	2.113	2.283	8.569
[4,5] ; [6,7]	0.052	50.700	2.326	2.670	21.175	0.061	50.700	2.388	2.791	21.441	0.054	50.150	2.209	2.544	13.637	0.056	50.450	2.584	2.627	9.186
[5,6] ; [3,4]	1.000	99.950	0.016	0.022	0.368	1.000	99.850	0.035	0.029	0.275	1.000	98.900	0.014	0.037	0.243	0.997	97.050	0.025	0.042	0.374
[5,6] ; [4,5]	0.997	98.850	0.046	0.081	0.568	0.997	98.850	0.068	0.080	0.621	0.994	98.200	0.045	0.080	0.473	0.990	96.950	0.051	0.088	0.600
[5,6] ; [5,6]	0.744	77.850	0.278	0.428	0.771	0.774	83.200	0.316	0.441	1.075	0.691	81.150	0.279	0.405	1.523	0.692	84.900	0.283	0.437	2.256
[5,6] ; [6,7]	0.152	55.400	1.091	1.415	28.359	0.136	56.150	1.152	1.508	28.414	0.137	53.750	1.055	1.358	20.042	0.126	57.900	2.133	2.339	15.443
[5,6] ; [7,8]	0.064	49.450	2.328	2.628	31.343	0.063	48.950	2.387	2.743	31.558	0.061	51.300	2.198	2.491	21.902	0.058	49.300	2.559	2.637	16.035
[6,7] ; [3,4]	1.000	99.750	0.013	0.015	0.382	1.000	99.700	0.035	0.028	0.803	1.000	99.050	0.012	0.035	1.547	0.998	97.550	0.022	0.038	2.050
[6,7] ; [4,5]	1.000	99.800	0.016	0.022	0.369	1.000	99.350	0.037	0.032	0.268	1.000	98.700	0.014	0.038	0.240	0.998	97.550	0.024	0.041	0.378
[6,7] ; [5,6]	0.997	99.000	0.046	0.084	0.569	0.997	99.100	0.068	0.083	0.620	0.992	97.450	0.046	0.082	0.465	0.985	96.150	0.052	0.092	0.609
[6,7] ; [6,7]	0.764	77.300	0.281	0.439	0.774	0.774	82.850	0.319	0.454	1.073	0.740	80.800	0.281	0.414	1.522	0.726	86.650	0.287	0.446	2.268
[6,7] ; [7,8]	0.143	55.300	1.085	1.447	39.863	0.140	57.150	1.139	1.531	39.953	0.158	54.300	1.046	1.388	29.785	0.104	57.650	2.081	2.367	24.028
[6,7] ; [8,9]	0.054	50.450	2.272	2.623	43.549	0.053	49.400	2.317	2.753	43.716	0.055	50.550	2.144	2.492	32.165	0.055	50.600	2.446	2.537	24.991
[7,8] ; [3,4]	1.000	99.800	0.013	0.014	1.623	1.000	99.400	0.037	0.029	3.067	0.999	98.450	0.012	0.036	4.799	0.994	96.800	0.021	0.037	5.746
[7,8] ; [4,5]	1.000	99.850	0.013	0.015	0.381	1.000	99.650	0.037	0.029	0.799	1.000	98.700	0.012	0.036	1.533	0.998	97.050	0.022	0.039	2.020
[7,8] ; [5,6]	1.000	99.650	0.016	0.022	0.372	0.999	99.350	0.036	0.031	0.268	0.999	98.350	0.014	0.037	0.239	0.997	96.950	0.024	0.042	0.373
[7,8] ; [6,7]	0.999	99.050	0.047	0.083	0.584	0.998	98.850	0.070	0.085	0.630	0.997	97.900	0.046	0.082	0.476	0.989	96.650	0.051	0.089	0.597
[7,8] ; [7,8]	0.745	76.950	0.273	0.427	0.756	0.742	81.450	0.314	0.439	1.063	0.676	79.550	0.275	0.403	1.520	0.638	84.600	0.280	0.433	2.272
[7,8] ; [8,9]	0.139	54.550	1.090	1.401	53.454	0.140	57.700	1.156	1.504	53.525	0.141	54.900	1.058	1.358	41.823	0.098	56.700	2.077	2.317	34.326
[7,8] ; [9,10]	0.055	50.850	2.278	2.595	57.280	0.053	50.900	2.352	2.721	57.129	0.056	49.950	2.157	2.453	44.185	0.063	50.650	2.528	2.622	35.398
[8,9] ; [3,4]	1.000	99.600	0.013	0.014	4.711	1.000	99.600	0.039	0.030	7.317	1.000	98.600	0.012	0.036	10.009	0.996	97.400	0.022	0.038	11.332
[8,9] ; [4,5]	1.000	99.950	0.013	0.014	1.611	1.000	99.700	0.034	0.030	3.049	1.000	98.300	0.012	0.036	4.773	0.998	96.700	0.022	0.037	5.661
[8,9] ; [5,6]	1.000	99.650	0.013	0.015	0.392	1.000	99.450	0.035	0.029	0.805	1.000	99.450	0.012	0.035	1.544	0.996	97.800	0.021	0.040	2.029
[8,9] ; [6,7]	1.000	99.700	0.016	0.022	0.373	1.000	99.450	0.039	0.033	0.268	0.999	98.550	0.015	0.041	0.242	0.996	96.950	0.024	0.045	0.378
[8,9] ; [7,8]	1.000	99.050	0.047	0.083	0.567	0.997	99.200	0.070	0.083	0.633	0.995	97.950	0.046	0.082	0.474	0.991	97.300	0.052	0.092	0.606
[8,9] ; [8,9]	0.749	76.000	0.284	0.423	0.753	0.743	81.050	0.319	0.443	1.077	0.694	80.350	0.282	0.398	1.518	0.635	84.450	0.288	0.429	2.252
[8,9] ; [9,10]	0.130	54.600	1.097	1.431	68.993	0.142	57.000	1.163	1.525	68.923	0.123	54.000	1.064	1.379	55.608	0.105	58.050	2.055	2.324	46.914
[9,10] ; [3,4]	1.000	99.650	0.014	0.014	9.936	1.000	99.400	0.043	0.033	13.665	1.000	98.200	0.013	0.036	17.290	0.998	96.750	0.021	0.039	19.036
[9,10] ; [4,5]	1.000	99.850	0.013	0.014	4.742	1.000	99.250	0.039	0.031	7.349	0.999	98.200	0.014	0.036	10.059	0.995	97.200	0.023	0.038	11.380
[9,10] ; [5,6]	1.000	99.650	0.013	0.014	1.609	0.999	99.400	0.038	0.029	3.032	0.999	98.400	0.011	0.035	4.749	0.997	96.450	0.022	0.037	5.669
[9,10] ; [6,7]	1.000	99.800	0.013	0.015	0.386	1.000	99.550	0.037	0.031	0.809	1.000	98.600	0.012	0.036	1.531	0.998	97.800	0.022	0.039	2.044
[9,10] ; [7,8]	1.000	99.550	0.016	0.022	0.385	1.000	99.250	0.038	0.033	0.279	0.999	98.100	0.015	0.039	0.235	0.994	96.900	0.025	0.043	0.369
[9,10] ; [8,9]	1.000	99.500	0.048	0.082	0.568	0.999	99.300	0.070	0.081	0.625	0.996	98.550	0.047	0.081	0.477	0.993	97.100	0.054	0.089	0.622
[9,10] ; [9,10]	0.738	76.750	0.282	0.437	0.766	0.718	80.800	0.319	0.453	1.063	0.685	79.600	0.282	0.413	1.528	0.691	84.900	0.290	0.446	2.275

Table S2 | Means and medians of distributions of parameter prediction errors of CLOUDe and LRT.

Method	Redundant mean	Redundant median	Unique mean	Unique median
NN	0.010	0.080	0.020	0.100
XGB	-0.005	0.001	0.040	0.040
RF	-0.030	-0.160	0.050	-0.060
SVM	0.002	-0.080	0.030	-0.030
LRT	0.190	-0.110	0.690	0.320

Table S3 | Statistically significant results of DAVID analysis for "redundant" genes.

Term code	Term	p-value*	Genes
GO:0009897	external side of plasma membrane	2.08×10^{-2}	*CG32146*, *CG14499*, *CG8343*, *CG14500*
GO:0016746	transferase activity, transferring acyl groups	3.63×10^{-2}	*CG17597*, *CG17320*, *CG4753*, *CG4729*
GO:0016485	protein processing	4.73×10^{-2}	*CG5527*, *CG14526*, *CG14527*, *CG8358*

* After Benjamini-Hochberg procedure.

Table S4 | Statistically significant results of DAVID analysis for "unique" genes.

Term code	Term	*p*-value*	Genes
GO:0016579	protein deubiquitination	4.63×10^{-5}	*CG3781*, *CG12231*, *CG7222*, *CG1950*, *CG3431*
GO:0004843	thiol-dependent ubiquitin-specific protease activity	1.28×10^{-4}	
GO:0005741	mitochondrial outer membrane	2.52×10^{-4}	*CG8330*, *CG1134*, *CG5395*, *CG12157*, *CG4701*

* After Benjamini-Hochberg procedure.

Table S5 | Training parameters, error metrics, and optimal settings for CLOUDe NN, XGB, SVM, and RF architectures.

Method	Training hyperparameters	Error metric	Lowest error for
NN	Number of layers, $L \in \{0,1,\ldots,5\}$<br>$\log_{10}(\lambda) \in [-12, -3]$, 25 evenly distributed values<br>$\gamma \in [0, 1]$, 11 evenly distributed (for ridge $\gamma = 0$ and for lasso $\gamma = 1$)<br>Batch size = 5000 observations per epoch<br>Number of epochs = 500	Five-fold cross validation	Classification:<br>$L = 2$<br>$\lambda \approx 1.778 \times 10^{-4}$<br>$\gamma = 0.9$<br>Regression:<br>$L = 3$<br>$\lambda \approx 7.498 \times 10^{-5}$<br>$\gamma = 0.9$
XGB	Booster = "gbtree"<br>Maximum depth of trees, $D \in \{1,2,\ldots,6\}$ (used as parameter "max_depth" of xgboost package)<br>Learning rate, $\eta \in [0.01, 3]$, 4 evenly distributed values (used as parameter "eta" of xgboost package)<br>$\gamma \in [0, 1]$, 11 evenly distributed values*<br>$\log_{10}(\lambda) \in [-12, -3]$, 25 evenly distributed values*<br>* $\lambda(1-\gamma)$ and $\lambda\gamma$ were used as the values for parameters "lambda" and "alpha" of the xgboost package, respectively<br>Maximum number of iterations, "nrounds" = 500<br>Early stopping after 50 rounds without loss minimization	Five-fold cross validation	Classification:<br>$D = 4$<br>$\lambda \approx 2.371 \times 10^{-9}$<br>$\gamma = 0.2$<br>$\eta \approx 0.2003$<br>Regression:<br>$D = 4$<br>$\lambda \approx 7.498 \times 10^{-11}$<br>$\gamma = 0.8$<br>$\eta \approx 0.2003$
SVM	Radial basis kernel with kernel width γ<br>Regularization parameter $\log_{10}(C) \in [-3, 3]$, 7 evenly distributed values<br>$\gamma \in [0.001, 5]$, 11 evenly distributed values	Five-fold cross validation	Classification:<br>$\log_{10}(C)$ = -3<br>$\gamma = 2.0006$<br>Regression:<br>$\log_{10}(C)$ = 1<br>$\gamma = 1.0008$
RF	Number of decision trees, $T = 500$	Chosen to be sufficiently large where out-of-bag errors plateaued in initial experiments	Same number of trees for classification and regression predictors

Table S6 | Details of the NN architecture of CLOUDe for all values of *L*.

L	Hidden layer: units (activation function)	Output layer (activation function)
0	None	Classifier: 2 units (softmax)<br><br>Regression: 3m units (linear),<br>where m = number of conditions
1	Layer 1: 256 units (ReLU)	
2	Layer 1: 256 units (ReLU)<br>Layer 2: 128 units (ReLU)	
3	Layer 1: 256 units (ReLU)<br>Layer 2: 128 units (ReLU)<br>Layer 3: 64 units (ReLU)	
4	Layer 1: 256 units (ReLU)<br>Layer 2: 128 units (ReLU)<br>Layer 3: 64 units (ReLU)<br>Layer 4: 32 units (ReLU)	
5	Layer 1: 256 units (ReLU)<br>Layer 2: 128 units (ReLU)<br>Layer 3: 64 units (ReLU)<br>Layer 4: 32 units (ReLU)<br>Layer 5: 16 units (ReLU)	
